# *Coxiella burnetii* in Dromedary Camels (*Camelus dromedarius*): A Possible Threat for Humans and Livestock in North Africa and the Near and Middle East?

**DOI:** 10.3389/fvets.2020.558481

**Published:** 2020-11-05

**Authors:** Christian A. Devaux, Ikram Omar Osman, Matthieu Million, Didier Raoult

**Affiliations:** ^1^Aix-Marseille Univ, IRD, APHM, MEPHI, IHU-Méditerranée Infection, Marseille, France; ^2^CNRS, Marseille, France; ^3^Faculty of Sciences Ben-Ben-M'Sik, University Hassan II, Casablanca, Morocco

**Keywords:** *Coxiella burnetii*, dromedary camel (*Camelus dromedarius*), zoonoses awareness, epidemiology, human—animal coexistence

## Abstract

The “One Health” concept recognizes that human health is connected to animal health and to the ecosystems. *Coxiella burnetii*–induced human Q fever is one of the most widespread neglected zoonosis. The main animal reservoirs responsible for *C. burnetii* transmission to humans are domesticated ruminants, primarily goats, sheep, and cattle. Although studies are still too sparse to draw definitive conclusions, the most recent *C. burnetii* serosurvey studies conducted in herds and farms in Africa, North Africa, Arabian Peninsula, and Asia highlighted that seroprevalence was strikingly higher in dromedary camels (*Camelus dromedarius*) than in other ruminants. The *C. burnetii* seroprevalence in camel herds can reach more than 60% in Egypt, Saudi Arabia, and Sudan, and 70 to 80% in Algeria and Chad, respectively. The highest seroprevalence was in female camels with a previous history of abortion. Moreover, *C. burnetii* infection was reported in ticks of the *Hyalomma dromedarii* and *Hyalomma impeltatum* species collected on camels. Even if dromedary camels represent <3% of the domesticated ruminants in the countries of the Mediterranean basin Southern coast, these animals play a major socioeconomic role for millions of people who live in the arid zones of Africa, Middle East, and Asia. In Chad and Somalia, camels account for about 7 and 21% of domesticated ruminants, respectively. To meet the growing consumers demand of camel meat and milk (>5 million tons/year of both raw and pasteurized milk according to the Food and Agriculture Organization) sustained by a rapid increase of population (growth rate: 2.26–3.76 per year in North Africa), dromedary camel breeding tends to increase from the Maghreb to the Arabic countries. Because of possible long-term persistence of *C. burnetii* in camel hump adipocytes, this pathogen could represent a threat for herds and breeding farms and ultimately for public health. Because this review highlights a hyperendemia of *C. burnetii* in dromedary camels, a proper screening of herds and breeding farms for *C. burnetii* is urgently needed in countries where camel breeding is on the rise. Moreover, the risk of *C. burnetii* transmission from camel to human should be further evaluated.

## Introduction

Q fever is a neglected zoonotic disease caused by bacteria (1, 2). It is generally admitted that clones of *Coxiella burnetii*, the etiologic agent of Q fever, circulate in wildlife and infects domestic ruminants. Very few bacteria are required to initiate the infection process (3). Usually, humans become infected through the aerosol route during contact with *C. burnetii*–positive domestic animals or their products (2, 4). Infection of humans concerns first the farmers and other professionals that have contacts with animals (e.g., veterinarians), but epidemics have been reported in other social groups. *C. burnetii* is a strict intracellular Gram-negative bacterium entering different cell types with progressive variation in the structure of its lipopolysaccharide (LPS): a smooth LPS for the virulent phase I and a rough LPS for the less virulent phase II (5–7). For symptomatic cases, human Q fever usually occurs 2 to 6 weeks after bacterial exposure (8, 9). The symptomatic primo-infection (10–60% of cases), called acute Q fever, is characterized by high fever, headache, myalgia, pneumonia, and hepatitis (2, 10). It usually resolves spontaneously in a few weeks. When Q fever is suspected, confirmation is provided by serological diagnosis based on anti–*C. burnetii* immunoglobulin (Ig) detection. An IgG anti–phase II Ig titer above 1:200 and an IgM titer above 1:50 are considered significant for the diagnosis of acute Q fever. Sometimes the symptoms do not resolve (about 5% of cases) and settle in a persistent way mainly in the heart valve and vascular wall but also lymph node, and bone (11). Other disorders can be associated with persistent infections, including lung diseases, hepatitis, and B-cell lymphoma (12, 13).

Regarding domestic ruminants, *C. burnetii* is responsible for epizooties with increased morbidity and mortality in livestock. It has long been considered that sheep, goats, and cattle were the main domestic source of *C. burnetii* worldwide among ecosystems in which *C. burnetii* clones circulate. Although *C. burnetii* has been classified as a notifiable animal disease by the World Organization for Animal Health (14), notifications concern only a subgroup of domestic animal species and ignore the bacteria dynamics in different ecosystems. Among ruminant species, camels are present in the countries of the Southern coast of Mediterranean basin but absent from countries of the Northern coast. The fact that some Southern countries practice intensive camel breeding, that a high percentage of these animals are carriers of anti–*C. burnetii* Ig, that *C. burnetii* was found in camels raw milk, and that camel ticks carries the bacteria must make us question our global perception of the mode of *C. burnetii* transmission in the Southern coast countries of Mediterranean basin. This review compiles data from the literature regarding the countries around the Mediterranean basin and the Arabic peninsula where camel breeding is practiced and highlights that the potential role of camels as a bacterial reservoir in the transmission of *C. burnetii* to humans should be considered.

## Human Q Fever Is Found on Six of Seven Continents: An Epidemiological Overview

**Figure 1:**
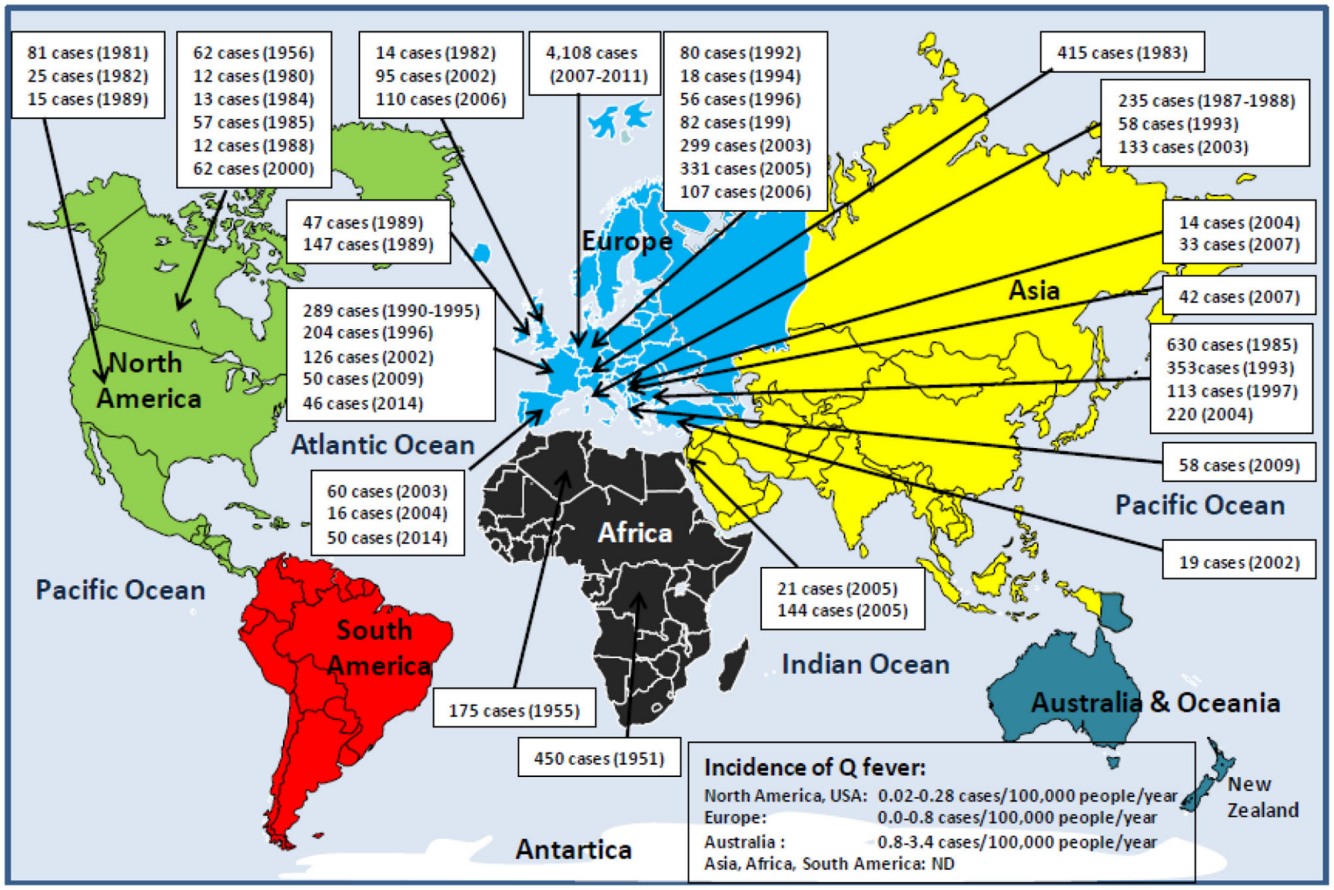
Schematic representation of human Q fever epidemiology around the world. With the exception of Antarctica and New Zealand, Q fever is a global zoonosis present in North America, South America, Europe, Asia, Africa, and Australia/Oceania. The clinical manifestation of Q fever in human is usually an undifferentiated febrile illness. Q fever was described for the first time in humans in 1937 by Burnet, who investigated several cases of Australian abattoir workers suffering from undifferentiated febrile illness (15, 16). During the Second World War (1941–1944), the Q fever disease was reported among German soldiers stationed in the Balkans, Southern Italy, Corsica, in English and American allied troops in Central Italy, and soldiers in Crimea and Ukraine. That is why the disease has had many synonyms: Olympus fever, Crimean fever, flu Balkan flu, Cretan pneumonia, Euboea fever, fever of the 7 days, or Derrick and Burnet's disease (17). The causative agent of the disease first identified by Cox in the United States, and formerly named *Rickettsia diasporica*, was definitively renamed *Coxiella burnetii* (18–20). The figure illustrates the history of the major human epidemics of Q fever (outbreaks >10 linked cases) from 1950 (when the Third World Health Assembly passed a resolution calling for study of the prevalence of Q fever throughout the world) to the present day. Although *C. burnetii* infection has been classified as a notifiable animal disease by the World Organization for Animal Health, OIE (14), the lack of mandatory reporting of human Q fever cases in most countries, the predominance of asymptomatic forms, the clinical polymorphism, and the difficulty of diagnosis are likely to lead to a significant underestimation of the true incidence of the disease in humans. In Europe where the ECDC carries out a regular epidemiological surveillance, only 1,023 of 4,245 Q fever cases confirmed during the 2013–2017 period were reported by the European countries (21). It is impossible to evaluate the number of cases for the Asian and African continents. ND, not determined.

As far back as 1950, the third World Health Assembly was aware of the potential danger of Q fever to public health and passed a resolution calling for study of the *C. burnetii* prevalence worldwide. Since then, numerous epidemiological studies have shown that human Q fever is found almost everywhere on the planet, with only exceptions of the Antarctica continent and New Zealand. Although seroprevalence data are available for most countries, it can be considered that the true incidence of Q fever in humans is largely underestimated because (i) Q fever is a neglected infectious disease; (ii) there is a predominance of asymptomatic forms; (iii) Q fever is rarely a notifiable disease, and there is a lack of mandatory reporting (e.g., Q fever become a notifiable illness in 1977 and 1978 in Australia and the Netherlands, respectively, and a reportable disease in 1999 in the United States and Japan); and (iv) several local reports written in languages other than English remain ignored (Figure 1).

On the North American continent, between 1946 and 1977, a total of 1,169 human Q fever cases were reported in the United States (22–25). Then, from 1978 to 2016, about 200 cases of human Q fever were reported annually, a mean of about 0.25 cases/100,000 inhabitants/year (cases/100 kI/y), with a seropositivity of 3.1% in adult populations rising 22% among the veterinarians (26–29). In Canada, *C. burnetii* in humans was first reported in 1952 (30, 31). In 1956, an outbreak with 62 human cases was reported in people working in a slaughterhouse, then individual cases in 1960 and 1966, followed by several outbreaks between 1975 and 1989 (32–34). In Central America, Q fever cases were reported in El Salvador and Mexico (35, 36). In the South American continent, during the 2013–2014 outbreak of dengue in Brazil, *C. burnetii* was identified in 3.3% of patients (37). Q fever cases were also reported in most South American countries (38–41). A very high incidence was also observed in the French Guiana in 2005 (150 cases/100 kI/y) (42).

On the European continent, more than 1,000 human Q fever cases were reported among soldiers in the Balkans in the early 1940s. Large-scale outbreaks were documented over recent decades, and serosurveys suggest a seroprevalence between 2 and 14% of the population (18, 43). The disease is endemic in Germany with 27 to 100 Q fever annual cases (incidence is 0.08–0.14 cases/100 kI/y), and 40 Q fever outbreaks documented (44, 45). In the United Kingdom, from 1975 to 1996, between 67 and 169 Q fever annual cases were reported (incidence of 0.15–0.35 cases/100 kI/y), including eight outbreaks (46–49). In 1983, a large outbreak of 415 human Q fever cases was reported in Switzerland (50). Until 2007, in the Netherlands, 5 to 16 Q fever cases were reported annually (51, 52). In 2007–2010, a large human outbreak with an estimated 44,000 people infected in 3 years was reported, among which were 4,108 cases of Q fever (53–56). In Portugal, the average frequency of Q fever is 0.1 case/100 kI/y, yet it is likely underestimated (57, 58). In the Spanish Canary Islands, a seroprevalence of infection by *C. burnetii* in humans of 36% was reported during an outbreak of Q fever (59). In France, the seroprevalence for anti–*C. burnetii* Ig was estimated 5/100 kI/y (60). In Bulgaria, from 1949 to 1993, more than 20 Q fever outbreaks occurred with three major outbreaks between 1982 and 1985, and next in 1993 and 1997 (61–64). In the late 2010s, 139 Q fever cases were reported (incidence of 0.27 cases/100 kI/y) (65). In Slovakia, a seroprevalence of 3% was estimated for the period before 1993 (63). According to OIE, between 1996 and 2001, eight Q fever cases were reported in Hungary, 26 cases in Ukraine, and 138 cases in Yugoslavia. In Russia, an outbreak in Leningrad affected 48 people in 1957 and between 1957 and 1995 up to 11,058 Q fever cases were reported (66–68).

On the Asian continent, 1% of patients hospitalized for infectious endocarditis and 14.6% of patients hospitalized for acute febrile illness/pneumonitis in India were infected by *C. burnetii* (69, 70). In Iran, 4.2% of patients with febrile illness and 18.1% of butchers and slaughterhouse workers carried anti–*C. burnetii* Ig (71, 72). In China, Q fever was initially reported in 1950 in a patient with pneumonia, and then in the 1960s, five outbreaks of Q fever occurred in abattoir workers, stockyard men, and troops (73, 74). Between 1989 and 2013, human Q fever cases were reported in people from 15 provinces in China and 4% of patients with infectious endocarditis suffered from Q fever (75, 76). In Japan, serosurveys indicated the presence of anti–*C. burnetii* Ig in 16.5% of human serum samples collected between 1978 and 1991 (77, 78). Since 1999, 7 to 46 Q fever cases were reported annually (79–82). In the Arabian Peninsula, the presence of *C. burnetii* in humans was reported in 1968, and a recent serological analysis detected *C. burnetii* Ig in 35.2% of patients with pyrexia of undetermined cause (83–85). In Qatar, Q fever data are rare, yet a seroprevalence of 2.1% was found in US soldiers deployed in this country (86).

On the Australian/Oceanian continent, since the first description of Q fever in 1937, the disease has continued to be endemic in Australia (87). Between 1977 and 1994, 202 to 860 cases were reported annually (incidence 3.11–4.99 cases/100 kI/y), despite a vaccine is recommended to farmers since 1989 (88–91). New Zealand is considered free from Q fever.

On the African continent, outbreaks of Q fever were reported in the early 1950s, but the disease remained neglected and underestimated (92–95). In Rwanda, an outbreak with 450 Q fever cases and 40 deaths linked to *C. burnetii* was reported (96). In Western Africa, seroprevalence in human was found to be 5% in rural Western Ivory Coast, 8% among nomads in rural Northern Burkina Faso, and 6–9% of patients hospitalized for pneumonia in Cameroun (97–101). *C. burnetii* was incriminated in 10% of children with non-malaria febrile illness (NMFI) in Niger, 8% in Gambia, and 17% in Ghana (102–104). Q fever is responsible for 2 to 9% of human hospitalization for NMFI in Middle, Central, and West Africa (105–107). In Eastern Africa, *C. burnetii* seroprevalence was reported to be 5% in pregnant women (108). A serological testing carried out in Kenya in 2016 indicated that 2.5% of people were seropositive for *C. burnetii* (109). In South Africa, a recent study reported that 38% of NMFI patients and 61% of workers in contact with camels (farmers, herders, and veterinary) carried anti–*C. burnetii* Ig (110).

## Human Q Fever Epidemiology Around the Mediterranean

**Figure 2:**
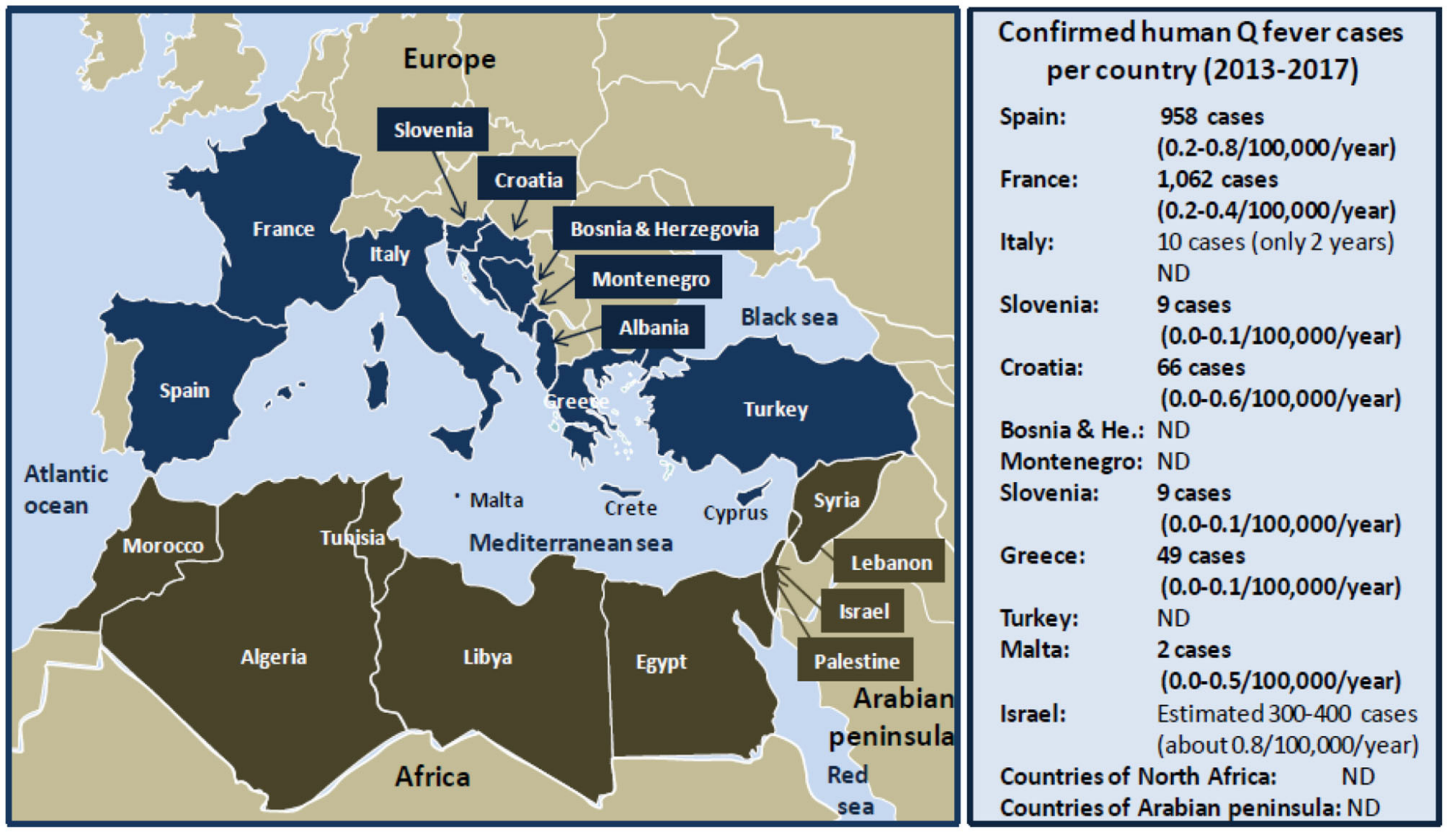
Schematic representation of human Q fever around the Mediterranean. **Left** panel: map of the Mediterranean basin. The Mediterranean Sea is bordered by 22 riparian countries. The countries of the Northern Mediterranean coast are represented in dark blue, and the countries of the Southern Mediterranean coast are represented in tanned brown. **Right** panel: confirmed human Q fever cases per country during the period 2013 to 2017 according to the ECDC (21). Q fever surveillance report, 2017. Values between brackets indicate the average number of Q fever cases per 100,000 inhabitants per year over the 5 years period. Regarding Italy, no data were available for the years 2013, 2014, and 2015. The number of cases of Q fever for Israel over the period 2013 to 2017 was extrapolated from the data published by Yarrow and colleagues (111). ND, not determined. Human Q fever occurs mostly in the form of sporadic cases. Sometimes outbreaks of Q fever were reported in humans. The main epidemics of Q fever described during the last 40 years in people living on the Northern coast of the Mediterranean basin are as follows: 2003 (60 cases), 2004 (16 cases), 2014 (50 cases) in Spain; 1992 (40 cases), 1996 (204 cases), 2002 (126 cases), 2009 (50 cases), 2014 (46 cases) in France; 1993 (58 cases), 2003 (133 cases) in Italy; 2007 (33 cases) in Slovenia; 2004 (14 cases) in Croatia; 2007 (42 cases) in Albania; 2009 (58 cases) in Greece; and 2002 (19 cases) in Turkey. Human Q fever outbreaks are poorly documented concerning the countries of the Southern Mediterranean Sea coast. An epidemics of Q fever was described in 1955 in Algeria with 175 cases.

The Mediterranean is bordered by 22 riparian countries (Figure 2) including the following:

- in the North: Spain, France, Monaco principality, Italy, Slovenia, Croatia, Bosnia Herzegovina, Montenegro, Albania, Greece, Turkey, Malta, and Cyprus; and
- in the South: Syria, Lebanon, Israel, Palestine, Egypt, Libya, Tunisia, Algeria, and Morocco.

On the Northern Mediterranean coast, Q fever is endemic in countries of the South Europe (Spain, France, Italia). From 1981 to 1998, more than 600 cases of Q fever were reported in Spain, most of which sporadic, except three outbreaks in 1989 (5 cases), 1990 (30 cases), and 1998 (14 cases) (112–116). Between 2000 and 2009, hundreds of Q fever cases were reported, most of which sporadic with an epidemic episode in the Asturias with 60 cases in 2003, and two outbreaks (16 and 22 cases, respectively) in Madrid (117–119). During the 2011–2015 period, 50 human Q fever cases were reported in Vizcaya and among 155 subjects with febrile illness from Galicia, 25% (39/155) were diagnosed with Q fever, and 6 patients died (120, 121). In France, Q fever was first observed in 1948 among slaughterhouse workers in Strasbourg. Between 1949 and 1953, cases were reported in Paris, in the region of Lyon and Northwestern (122). An intrafamily Q fever outbreak was induced by infected pigeons (18). The seroprevalence in humans can go up to 30% in the Alps rural populations (123). In the South of France, 5 to 8% of cases of endocarditis are due to *C. burnetii*, and a retrospective analysis performed on 22,496 sera showed a seroprevalence of 7.8% (1,754/22,496) with 323 acute Q fever (124, 125). Between 1990 and 1996, three outbreaks (including 289 Q fever cases in Martigues and 204 cases in Briançon) were linked to meet with infected sheep or goat, animal carcasses, and/or consumption of unpasteurized milk (126, 127). In 2002, an outbreak of 126 human Q fever cases possibly contaminated by ovine livestock occurred in Chamonix (128). In 2009, an outbreak of 50 human cases of Q fever was reported in Cholet (129), and in 2014, an outbreak of 46 cases of Q fever occurred after people had visited a sheep farm (130). In Italy, Q fever emerged in the late 1940s with epidemic outbreaks, and then it became endemic with sporadic occurrence (131). However, an epidemic outbreak was reported in 1996 in the Vicenza region with 58 human cases after contact with infected sheep (132). In 2003, an outbreak of 133 human Q fever was reported in Como, the prison being mainly concerned with a prevalence of disease of 10.8% (59/547) in prisoners, 16.5% (37/224) in guards, and 3.2% (33/1,025) in the city residents (133).

In the countries of the North coast of the Adriatic sea (Slovenia, Croatia, Bosnia Herzegovina, Montenegro, Albania), several reports indicated the presence of the pathogen. A group of 33 veterinarians contracted Q fever during a training course in Slovenia in 2007 (134). In Croatia between 1985 and 2002, 155 acute Q fever cases were hospitalized in Split, and the annual mean incidence was 0.20–4,64 cases/100 kI/y (135, 136). In 2004, an outbreak of 14 Q fever cases occurred in Zadar linked to contacts with infected sheep (137). During the 2008–2010 period in Croatia, a *C. burnetii* seroprevalence study indicated that 27.5% (152/552) of febrile patients with prolonged cough showed anti–*C. burnetii* Ig, and 5.8% developed acute Q fever (138). In the 2000s, a Q fever outbreak was reported in Albania in a group of 115 Argentinean police officers who were exposed to contaminated dust from infected sheep during a United Nations mission in Prizren in the South Kosovo, among whom 42 showed clinical symptoms of Q fever (139).

Q fever occurred in Greece in 1946 possibly due to consumption of milk from infected ovines (140). A serosurvey performed in Northern Greece in 1990 showed that 4.7% (173/3,686) of patients with atypical pneumonia had anti–*C. burnetii* Ig. During a 2-year survey on children hospitalized in Athens, acute Q fever was diagnosed in 0.67% (8/1,200) of patients, and Q fever accounted for 2.9% of the cases with prolonged fever (141). In 2009, 58 cases of Q fever were reported in Northern Greece (142). The mean rate of Q fever during 2004 to 2012 was 0.033 cases/100 kI/y. *C. burnetii* is endemic in the island of Crete. A high seroprevalence (38.7%) of anti–*C. burnetii* Ig was found in humans living in Crete, and 98 cases of Q fever were reported between 1989 and 1993 (143). In addition, a study over a period of 6 years (1989–1995) confirmed that 4.6% (152/3,300) of patients suspected of infection had anti–*C. burnetii* Ig (144). More recently, another serosurvey found a seroprevalence of 48.7% (240/493) (145). *C. burnetii* is also present in the islands of Malta and Cyprus. In Cyprus, a serosurvey study that investigated serum samples from 547 people found that 5.3% contained anti–*C. burnetii* Ig, whereas a more recent study using a similar number of samples indicated a seroprevalence of 52.7% (146, 147).

Q fever is considered endemic in Turkey. A total of 191 human Q fever cases were documented before 1953, most of them being sporadic (148, 149). In 2002, 46 cases of febrile illness were reported around the Black Sea in Northern Turkey, 19 with confirmed acute Q fever (150). The search for anti–*C. burnetii* Ig in 83 veterinarians indicated that 7–8% of them had been exposed to *C. burnetii*. A serosurvey on blood donors in Ankara showed that anti–*C. burnetii* IgG was detected in 32.3% (194/601) (151). In 2009, an investigation of *C. burnetii* prevalence in a group of 407 healthy subjects living in North Turkey indicated that 8.1% (33/407) of them showed evidence of contact with *C. burnetii* and 5.4% (22/407) were symptomatic with 17 acute Q fever and 5 persistent forms (152). Recently, the case of a young woman with Q fever endocarditis was reported (153). A recent seroprevalence study performed in the Erzincan province in the Eastern Turkey showed the presence of anti–*C. burnetii* Ig in 8.7% (32/368) of people (154).

In the Maghreb countries (Morocco, Algeria, Tunisia, Libya), *C. burnetii* was found in the early 1950s (94). In Morocco, a seroprevalence study conducted in 1995 reported that anti–*C. burnetii* Ig was present in 1% (1/300) of sera samples from Casablanca and 18.3% (23/126) of samples from Fez citizens (155). In Algeria, the first detection of human Q fever dates back to 1948 with 172 cases (156, 157). In 1955, an outbreak of Q fever concerned 175 infected soldiers from a French battalion who was quartered in stables recently occupied by horses and sheep (158). In 1960, several Q fever cases were reported in Eastern Algeria (17). A study performed on children younger than 16 years in Hoggar indicated a seroprevalence of anti–*C. burnetii* Ig of 20% (159). The follow-up of a human cohort of infective endocarditis in Algiers in 2000–2003 found a *C. burnetii* seroprevalence of 3% (2/61 patients) (160). A *C. burnetii* seroprevalence of 15.5% (113/729) was reported in humans in the Wilaya of Setif, an agropastoral region (161). In recent years, a limited number of human cases of Q fever were reported in Algeria, and most cases occurred in the Northern part of the country (160, 162). In Tunisia, a study of samples from a cohort of blood donors collected in 1993 in Sousse and its rural surrounding areas reported that 26% (130//500) of subjects had antibodies against *C. burnetii* (163). Yet most of serosurveys performed in Tunisia between 1990 and 2008 suggest a seroprevalence of *C. burnetii* between 1 and 3% (164–167). Information is missing regarding the human prevalence of *C. burnetii* infections in Libya. The serological study of foreigners (Czechoslovak citizens) returning to their country after they had worked in Libya between 1984 and 1988 showed an anti–*C. burnetii* Ig in 48 people, and about half of them had clinical symptoms of Q fever (168).

In the Mashreq (Egypt, Jordan, Palestine, Lebanon, Syria), a seroprevalence for anti–*C. burnetii* Ig ranging from 3 to 32%, was reported. An early study reported anti–*C. burnetii* Ig in 14.3% (11/77) of sera samples from Egypt (169). A *C. burnetii* seroprevalence of 32% (285/883) was reported in humans living near the Nile River Delta (170). In North Sinai in 2006, anti–*C. burnetii* Ig was found in 5.3% (8/150) of patients with pyrexia of unknown origin and 3.3% (1/30) of healthy controls (171). Another study found a seroprevalence of 16.3% (15/92) in humans who lived in agricultural districts (172). A more recent (2016–2017) study in El Minya Governorate reported a seroprevalence of anti–*C. burnetii* IgG of 25.7% (9/35) in farmers (173). Besides a case of Q fever in a Belgian patient who developed the disease after a journey in Syria was reported (174), there is no information available regarding human seroprevalence of anti–*C. burnetii* Ig in the Palestinian, Lebanese, Jordanian, and Syrian populations.

Q fever is endemic in Israel. Between 1981 and 1990, 758 cases of Q fever were reported. A more recent series of 34 cases of endocarditis allowed estimating the annual incidence of Q fever at 3.5 cases per year, or 0.075 cases/100 kI/y, likely linked to infected ruminants exposure (18, 175). In 2005, two outbreaks of Q fever (21 cases in Haifa and 144 cases in a school in central Israel) were reported (176, 177). A recent retrospective study reported 16 pediatric cases of Q fever (178). Another study investigated a cohort of patients admitted to Tel Aviv, Haifa, Hadera, and Kfar Saba hospitals between 2006 and 2016 and confirmed 38 cases of Q fever on 205 patients (179).

## The Main Known Reservoirs of *Coxiella burnetii*: Cattle, Sheep, Goats, What Else?

Domestic ruminants are considered the principal reservoirs for *Coxiella burnetii* and are frequently incriminated as sources of Q fever outbreaks in humans who become infected following inhalation of aerosols containing particles loaded with the bacteria or bacteria that survive in a spore-like state (95, 180, 181). *C. burnetii* was sometimes found in other domestic animals such as poultry, cats, dogs, rabbits, and pigeons (182–186). Different *C. burnetii* genotypes circulate in wildlife including clones that are more likely to cross species barrier for infection of livestock and humans (187–190).

Former epidemiological studies performed on cattle showed that when imported into an area of endemic infection, 40% of uninfected cows became *C. burnetii* infected within 6 months (191). Although the animals can develop metritis and mastitis, in cattle farms the disease usually evolves subclinically (79). The different clinical manifestations of the disease can lead to late gestation abortions, fertility disorders, and premature delivery (192, 193). Up to 10^9^ bacteria per gram can be contained in the placenta from infected ruminants (194–196). *C. burnetii* shedding is higher in vaginal mucus and feces than milk in the first 3 weeks postabortion or postpartum (197).

On the Australian/Oceanian continent, Q fever is the most commonly reported notifiable zoonotic disease in Australia after food-borne pathogens (198). Australia became the first country to use ruminants' vaccination. In New Zealand, in 1993, a large study conducted on 2,181 cattle and 12,556 sheep concluded that the country was free from coxiellosis. On the North American continent, a serosurvey performed in 1964 revealed that Quebec had the highest seroprevalence of anti–*C. burnetii* Ig in bovine (39.6%) (199). Sheep and goat occupy only a minor segment of farm activities in Canada, and their seroprevalence was 6.7 and 10.5%, respectively (19). Decades ago in the United States, a seroprevalence of *C. burnetii* Ig study in farm animals showed the highest seroprevalence among goats (41.6%), followed by sheep (16.5%) and cattle (3.4%) (200). In Asia, a seroprevalence study in Iran indicated that 13.6% (45/330) of sheep had anti–*C. burnetii* Ig (201). The overall prevalence of anti–*C. burnetii* Ig in China was 15% (288/1,918) in cattle and 12% (176/1,440) in goats (75). A recent study of seroprevalence in goats from the Hubei province of China reported that 4.75% (55/1,157) of animal had anti–*C. burnetii* Ig (202). In Europe, the main sources of human infection by *C. burnetii* were ovine products (203, 204). In 1983, a large outbreak of human Q fever was reported in Switzerland after sheep transhumance, with 38% of the animals being positive for anti–*C. burnetii* Ig (50). Most of the human epidemics reported in Germany were related to handling of infected sheep products (24 outbreaks), to contact with cattle (6 epidemics) or livestock (4 epidemics), or to work in slaughterhouses (2 epidemics). In 2003, 299 people were infected when a sheep gave birth at a livestock market in Soest (205). The large Q fever epidemics reported in 2007–2010 in the Netherlands were probably associated with the increase in goat farming (e.g., 5,000 in 1985 and up to 375,000 in 2009) (206–208), and a very high number of infected females as suggested by the frequency (20%) of abortions (209). The retrospective investigation of the origin of this *C. burnetii* outbreak in humans revealed that *C. burnetii* was also found in dogs and horses, as well as in wild deer (210, 211). In Portugal, the frequency of exposure of ovine herds at *C. burnetii* seems to be increasing with possible impact on humans (212). On the African continent, a *C. burnetii* surveys of ovines indicated seroprevalences of 13% in Chad, 24% in Sudan, and 29% in Niger (105, 213, 214).

In the countries from the Northern coast of the Mediterranean basin, cattle, goats, and sheep are considered the major reservoir of *C. burnetii* related to human infections. Serological studies performed on livestock in Madrid indicated that up to 76.6% of goats and 8.8% of cattle had anti–*C. burnetii* Ig (215). The investigations in livestock revealed that in Northern Spain, 3% of ovine carried *C. burnetii* (216). Other investigations reported the highest *C. burnetii* seroprevalence for sheep (31.5%), followed by goat (22.4%) and cattle (5.6%), respectively (217), and 7.7% (80/1,039) of ticks (mainly *Hyalomma rufipes*) (218). Surveys carried out on 5,081 cattle abortion cases from four rural regions in France between 1993 and 1996 confirmed *C. burnetii* infection in 0.5% to 3.8% of cases, while suspected for an additional 2 to 16% of cases (219). Serosurvey of *C. burnetii* in ruminant in Sicily (Southern Italy) also showed a very high seroprevalence of 73.6% in farm sheep (220). A serosurvey in Slovenia indicated that 46% of cattle, 36% (36/100) of sheep, and 2.4% (17/701) of ticks (mainly *Ixodes ricinus*) were exposed to *C. burnetii*, and ticks found positive by polymerase chain reaction (PCR) were most commonly (5.09%) sampled from wild deer (221). A recent serosurvey performed on 1,970 serum samples collected from farm cattle in three regions of Bosnia and Herzegovina indicated that 8.8% of animals were exposed to *C. burnetii* (222). In Turkey, the prevalence of *C. burnetii* exposed animals varies widely with species and geographic location (223). In Cyprus, a serosurvey indicated that many farm animals had been in contact with *C. burnetii* including 48.2% of goats, 24% of bovines, and 18.9% of sheep, with an overall abortion rate in the livestock population of Cyprus at 2 to 5% (147, 224). Among a total of 622 cow abortions in Cyprus in 2008–2009, *C. burnetii* infection was documented in 57% (29/51) of the tested samples (225). In 2013, in Malta, a 6-month ban was imposed on the transfer of cattle between farms because of an outbreak of *C. burnetii* infection in nine goats in one farm and two human cases. Altogether, these data (Table 1) strengthen the hypothesis that human Q fever epidemics in the countries of the Northern coast of the Mediterranean basin found their origin in sheep and/or goats mainly.

**Table 1:** History of the main human Q fever epidemics in countries of the Northern coast of the Mediterranean Sea and identification of the zoonotic source of *C. burnetii*.

**Year**	**Country**	**Probable origin**	**No. of human Q fever cases**	**References**
1987–1988	Italy	Sheep	235	(226)
1990–1995	France	Sheep	289	(227)
1992	France	Goat	40	(126)
1993	Italy	Sheep	58	(228)
1996	France	Sheep	29	(227)
1996	France	Sheep	204	(229, 230)
1997	Bosnia	Sheep	26	(227)
2000	France	Goat manure	10	(227)
2000	France	Sheep manure	5	(227)
2002	France	Sheep	126	(128)
2002	Turkey	ND^a^	19	(150)
2003	Italy	Sheep and goats	133	(133, 231)
2003	Spain	ND	60	(119)
2004	Croatia	Sheep	14	(137)
2004	Spain	Sheep and goats	22	(118)
2005	Slovenia	Sheep	33	(134)
2007	France	Sheep	18	(203)
2009	France	ND	50	(129)
2009	Greece	ND	58	(142)
2014	France	Sheep	46	(130)
2014	Spain	ND	50	(120)

a *ND, not determined*.

In the countries from the Southern coast of the Mediterranean basin, the earliest investigations of *C. burnetii* in the ecosystem of Morocco indicated the presence of the bacteria in sheep, goat, cattle, camels, gerbil, and ticks (94). A recent serosurvey of cattle in the North-East state of Setif indicated a seroprevalence of 11.36% (77/678) in cows (232). A study indicated that ticks collected on camels (*Hyalomma dromedarii*) and bulls (*Hyalomma excavatum*) imported in Egypt from Sudan were infected with *C. burnetii* (233). Other studies found the presence of *C. burnetii* in livestock with a seroprevalence of 22.5 to 32.7% in sheep, 16.8 to 28.2% in goat, and 13 to 13.2% in cattle, respectively (171–173, 234). A large survey that included livestock from Western desert, Nile River Valley, and Delta region reported anti–*C. burnetii* Ig in 19.3% (162/840) of cattle, 8.9% (64/716) of sheep, and 6.8% (21/311) of goats (235). The *C. burnetii* surveys of cattle indicated seroprevalences of 16 and 10% to 29% in Tunisia and Algeria, respectively (236–239).

Regarding the different human epidemics of Q fever in the countries of the Southern coast of the Mediterranean basin, similar to what has been demonstrated for the countries of the North Mediterranean coast, it was assumed that the source of bacteria came from cows, sheep, and/or goats (124), although it may be partly wrong because of the presence of animal species endemic to these countries.

## *C. burnetii* Still Neglected In the Oie List of Zoonotic Pathogens From Dromedary Camels

**Figure 3:**
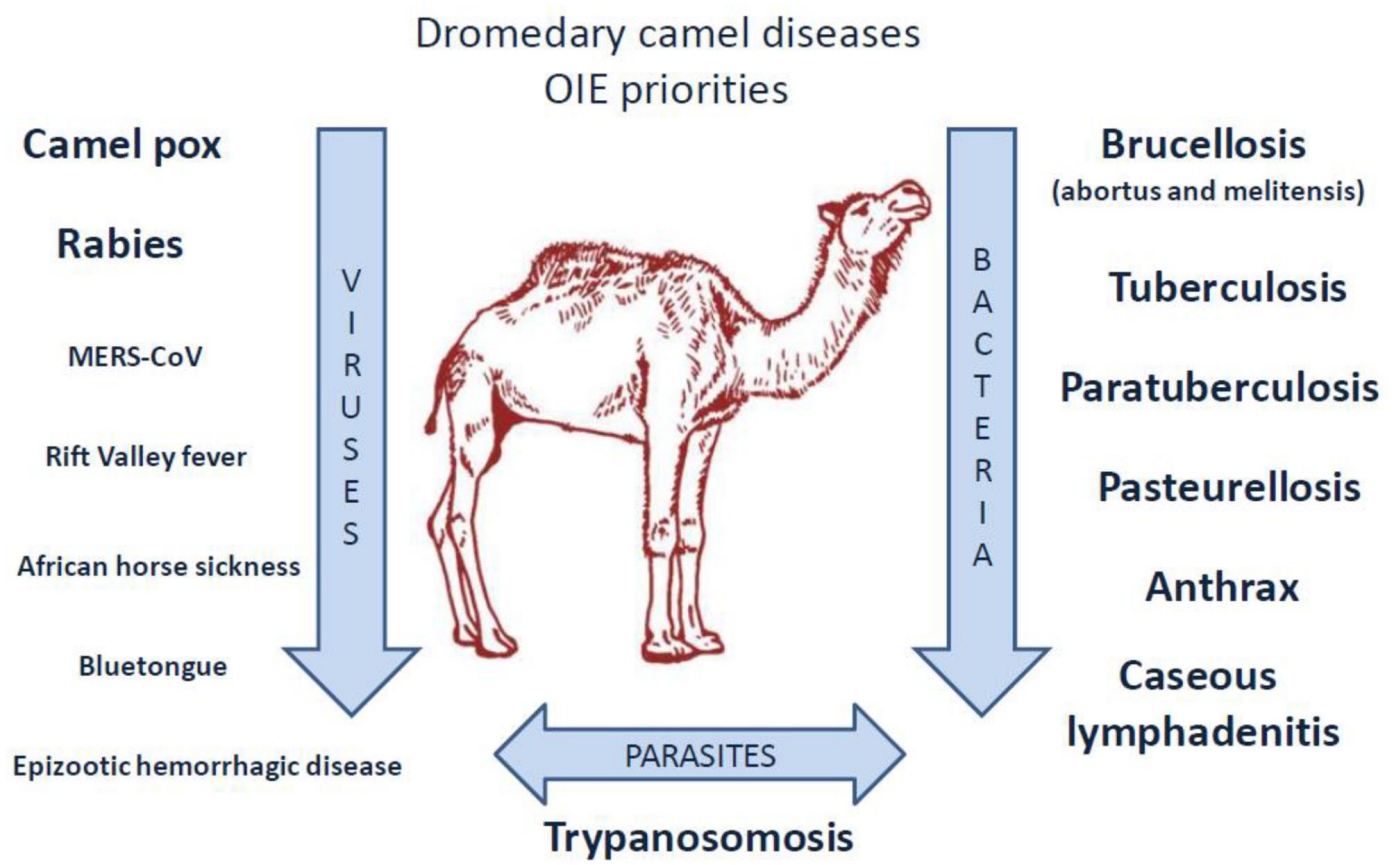
Priority diseases of camelids according to OIE (240). A list of diseases affecting camels and that appear to be priorities for the OIE to improve diagnostic capacity and establish guidelines for trade of camels and camel products was drawn up in 2014 by the OIE *ad hoc* Group on camel diseases. These experts divided the priority diseases into three groups: (1) significant diseases; (2) diseases for which camelids are potential pathogen carriers; (3) minor or non-significant diseases. Regarding the priority viral diseases (significant diseases), only camelpox and rabies were listed [foot and mouth disease (FMD) that concerns bactrian camels only, also belonged to the list]. The *ad hoc* Group classified MERS-CoV, Rift valley fever, and orbivirus-induced diseases (BT, AHS, EHD) among the diseases for which camelids are potential pathogen carriers (the bovine viral diarrhea that concerns the New World camelids also belonged to that list). Regarding bacteria, the *ad hoc* Group classified brucellosis, tuberculosis, paratuberculosis, anthrax, caseous lymphadenitis, and pasteurellosis in the significant diseases category. Trypanosomosis was classified in the significant parasitic diseases of camelids. It should be noted that coxiellosis is not mentioned in the lists of priority camel diseases for the World Organization for Animal Health, OIE. However, *C. burnetii* has been classified as a notifiable animal disease by this international office (14).

A listing of camel diseases considered a major threat, which ignored Q fever as “major threat” (although it appears as “notifiable disease”), was drawn by OIE in 2008 and updated in 2010 (240). Compared to other domestic species present on both sides of the Mediterranean, little is known about the pathogens that circulate in camel herds (241–244), probably due to a lack of international concern for camels (the earliest serosurveys were performed by biologists from Northern countries where camel is absent) (Figure 3). In the last decade, several camel diseases with overmortalities that occurred in African countries as well as Saudi Arabia attracted epidemiologists' curiosity. Today, OIE draws particular attention to camelpox and rabies viruses, to parasite-induced trypanosomosis, and to a few bacterial diseases including brucellosis, tuberculosis, paratuberculosis, pasteurellosis, anthrax, and caseous lymphadenitis.

Among the viral diseases affecting camels, camelpox is an economically important disease, notifiable to OIE (245–249). Camelpox is contagious in camel husbandry, and its mortality ranges from 0 to 40% (250, 251). This virus is a risk to the human population (252, 253), yet the disease can be prevented by vaccine and/or antiviral drugs such as cidofovir and ribavirin (254, 255). Rabies in camels is also observed in many countries from Africa, Arabian Peninsula, and Asia (256–262). Infection of camels was found preventable by canine inactivated rabies vaccine (263). Several other viruses able to infect camels are of concern for OIE. These viruses are the Rift valley fever (RFV), the Middle East respiratory syndrome coronavirus (MERS-CoV), the foot and mouth disease virus, the bluetongue virus, the epizootic hemorrhagic disease virus), the African horse sickness virus, and the Alkhurma hemorrhagic fever virus (AHFV) (264–297). In humans, the MERS-CoV and AHFV infections are known to be of high fatality rate (272–275, 298). Camel can also be infected by a number of other viruses (299–302).

Specific attention was drawn by OIE to *Trypanosoma* parasites (*Trypanosoma evansi, Trypanosoma vivax*), which can be the cause of abortion in camel herds (303–310). Other parasites and fungi also circulate in camel herds, including *Aspergillus fumigatus* considered responsible for the death of 40 racing camels in United Arab Emirates (UAE) during an outbreak of bronchopneumonia and gastroenteritis (311–313).

Because of the economic impact of brucellosis in ruminant herds (with losses on meat and milk sales due to abortion), special attention was focused on this disease in camels (314–321). In Saudi Arabia, whole herd vaccination using S19 or Rev1 vaccinal strains was reported to be successful for camel protection (322, 323). Finally, there is a public health concern linked to the risk of transmission to humans (324–327). Dromedary camel infection by *Mycobacterium tuberculosis* or *Mycobacterium bovis* was reported in several countries (328–341). Another mycobacterium, *Mycobacterium avium* subsp. *paratuberculosis*, is the causative agent of Johne disease that affects camels more severely than other ruminants (342–347). There is also concern by OIE for lung pseudotuberculosis abscesses, a frequent disease of camels, as well as pasteurellosis, anthrax, and plague (348–350). Cases of camel plague/*Yersinia pestis* were reported in Libya (351, 352), and human cases were described after consumption of meat from infected camels (353, 354). Obviously, camels are susceptible to a wide range of bacterial-induced diseases including mastitis (242, 355, 356), upper respiratory tract diseases (357–360), skin necrosis (361, 362), botulism (363), tetanus (364, 365), and diarrhea (299, 366–368).

## Camels: Another Animal Reservoir of *C. burnetii* Besides Ruminant Livestock and Wild Life?

Dromedary camels that are almost absent from the Northern countries of Mediterranean basin account for 3% of the domestic ruminant populations in the Southern countries of the Mediterranean basin (Table 2). Although this percentage is relatively low, it became necessary to revisit the epidemiological data and question the possible role of camels as a source of human Q fever. Sixty-five years ago, the presence of *C. burnetii* in camels was already reported (94). Regarding animal serosurvey, it is hazardous to directly compare the data obtained from one country to another by different laboratories under the format of a meta-analysis because of size of tested population, sample selection bias, and different technical methods of diagnosis. Yet, it remains intriguing that in most studies that included dromedary camels in the panels of ruminants tested for *C. burnetii* exposure or infection, the highest seroprevalence corresponded to dromedary camels ahead from the other ruminants (Table 3).

**Table 2:** National productions of farm ruminants and percentage of dromedary camels with respect to the total number of other domestic ruminants (cows, sheep, and goats).

**Ruminants**	**Cows**	**Sheep**	**Goats**	**Camels**	**% Camels/ruminants**
**Countries of the Southern coast of the Mediterranean basin**					
Morocco	3,364,000^a^	19,863,000	5,205,000	59,000	0.2%
Algeria	1,895,126	28,393,602	5,007,894	381,882	1.08%
Tunisia	627,614	6,536,762	1,205,526	237,005	2.75%
Libya	124,941	7,400,487	2,628,366	64,469	0.62%
Egypt	5,064,509	5,697,716	4,351,545	149,224	0.97%
Palestine	40,254	747,880	215,000	0	0%
Israel	543,311	519,640	89,720	5,530	0.47%
Lebanon	81,262	458,112	516,803	192	0.02%
Jordania	72,644	3,057,948	770,771	14,322	0.36%
**Other countries^b^**					
Chad	27,603,203	30,789,484	34,408,101	7,285,309	7.28%
Somalia	4,800,000	11,000,000	11,524,496	7,222,181	20.91%
Sudan	30,734,061	40,573,686	31,443,790	4,849,003	4.51%
Djibouti	299,954	468,732	514,462	70,965	5.24%
UAE	104,584	2,208,451	2,264,699	451,463	8.97%
Qatar	21,675	287,231	169,232	40,843	7.87%

a *Number of heads in herds and farms in 2017 according to FAO data (369)*.

b *Complementary data correspond either to the countries that are the largest producers of camels or to countries in which the ratio of camels per capita is the highest*.

**Table 3:** The seroprevalence of *Coxiella burnetii* in *Camelus dromedarius* camels compared to other ruminants.

**Country**	**% of camels**	**% of cattle**	**% sheep**	**% of goats**	**References**
Chad	80%	4%	33%	23%	(105)
Egypt	13.3%	ND	22.5%	16.8%	(171)
Egypt	ND	13%	33%	23%	(172)
Egypt	40.7%	19.3%	8.9%	6.8%	(235)
Iran	28.3%	13.3%	24.7%	31.9%	(370)
Kenya	20%	6%	13%	18%	(371)
Saudi Arabia	51.5%	30.7%	12.4%	34.0%	(372)
Sudan	64.3%	29.9%	ND	ND	(214)
China	NA	15%	ND	12%	(75)
Spain	NA	5.6%	31.5%	22.4%	(217)
USA	NA	3.4%	16.5%	41.6%	(200)

An investigation in Egypt that tested 200 camels for *C. burnetii* reported a seroprevalence of 66% (373). Another serosurvey two decades later reported anti–*C. burnetii* Ig in 40.7% of dromedary camels (mainly imported from Sudan), followed by cattle (19.3%), sheep (8.9%), and goat (6.8%) (235). In the study by Klemmer et al. the seroprevalence in camels from Aswan governorate bordering Sudan was 67.5%. This corroborates a study in Sudan that reported a seroprevalence of 64.3% (49/76) in camels and 29.9% in cattle (214). A recent study in Egypt reported that 4.5% (5/112) of camel sera were positive for anti–*C. burnetii* Ig, whereas a standard quantitative PCR found an overall prevalence of 15 to 19% (374). The only study that reported a higher seroprevalence in ovines than camels was performed in North Sinai, with the higher seroprevalence in sheep (22.5%), followed by goat (16.8%) and camels (13.3%), respectively (171). A serosurvey in Chad, highlighted that seroprevalence was the highest in dromedary camels (80%), followed by sheep (33%), goats (23%), and cattle (4%) (105). In Iran, on 167 camels that originated from 11 regions, a mean seroprevalence of 28.7% for *C. burnetii* (seropositivity ranging from 0 to 63.6%) was observed (375). A more recent study confirmed a seroprevalence of Q fever in camels of 28.3% in Iran, whereas for the other ruminants, the results were 31.9% in goats, 24.7% in sheep, and 13.3% in cattle (370). Studies in Saudi Arabia reported a seroprevalence around 50 to 60% of dromedary camels, with the most recent investigation reporting a seroprevalence of 51.5% in 489 camels from Saudi Arabia, whereas the seroprevalence was 34.0% in goats, 30.7% in cattle, and 12.4% in sheep, respectively (372, 376, 377). A serosurvey performed in Algeria revealed that 71.2% of dromedary camels had circulating *C. burnetii* Ig (378). A recent study conducted in Kenya confirmed that the highest seroprevalence was in dromedary camels (20%), followed by goats (18%), sheep (13%), and cattle (6%) (379). These results corroborate those from another study that reported a seroprevalence of 18.6% in camels (371).

**Figure 4:**
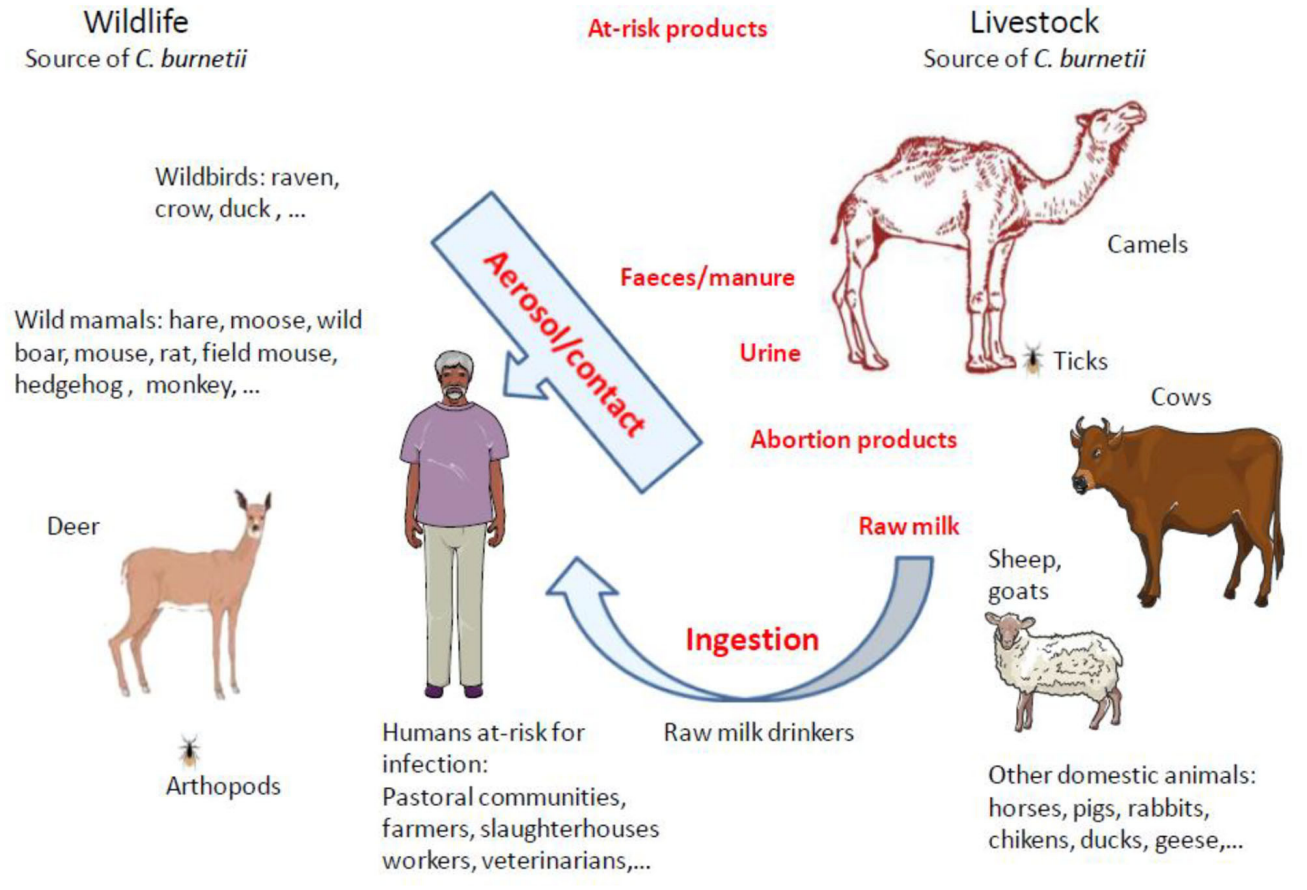
Schematic representation of animal reservoirs diversity and main routes of animal-to-human transmission of Q fever. Up to now, domestic ruminants were considered the principal reservoirs for *C. burnetii* and were frequently incriminated as sources of Q fever outbreaks in humans. However, in North Africa and Middle East were camels live, it was reported that most of the time the seroprevalence of anti–*C. burnetii* Ig was much higher in dromedary camels than in other ruminants. *C. burnetii* shedding is higher in vaginal mucus and feces than in milk. The infection in humans by *Coxiella burnetii* is mainly by inhalation of aerosols. It was evidenced that *C. burnetii* can be transmitted during arthropod blood-sucking. Among ticks, the *Hyalomma dromedarii* that colonize dromedari camels was found infected with *C. burnetii*. Human populations at risk of *C. burnetii* infection are pastoral communities, farmers, slaughterhouses workers, tanneries workers, veterinarians, or individuals handling infected livestock, especially animals giving birth. Raw milk drinkers are also at risk. With the increasing demand of milk and camel meat in urban areas, there is a potential threat for millions of people.

Many questions remain unanswered regarding the origins of the high prevalence of anti–*C. burnetii* Ig in dromedary camels (Table 4), the ways by which camels become infected, and their role as putative reservoir in transmission of *C. burnetii* to other ruminants and/or humans. It was reported that the preferred route of *C. burnetii* shedding by infected camels is feces (27.6% positive samples by PCR), followed by urine (23.8%) and milk (6.5%) (396) (Figure 4). A study on 534 healthy camels in Tunisia indicated that 44% (235/534) were seropositive to *C. burnetii*, and it reached 70% in female camels with a previous history of abortion (391). It is also possible that the high prevalence of anti–*C. burnetii* Ig in camels was related to infections by fleas or ticks during blood-sucking (397, 398). Among ticks, the *H. dromedarii* that colonize dromedary camels were found infected with *C. burnetii* (1, 233, 399). At every developmental stage of their life cycle, the *H. dromedarii* ticks feed only once, and their camel blood meal is sufficient for the molt to occur to the next stage (400). Female ticks deposit 10,000 to 20,000 eggs on the camel host body. Recently, a survey performed on dromedary camels and *H. dromedarii* ticks in Egypt found that 46% (52/113) of camels (27.1% of dromedary camels in Giza and 67.9% in Cairo) and 5.6% (10/177) of *H. dromedarii* ticks were positive for *C. burnetii* (383). In contrast, in the hot and dry regions of Southern Europe, other ticks such as *Dermacentor marginatus* were considered a possible vector of *C. burnetii* among ruminants (171, 401–403).

**Table 4:** The seroprevalence of *Coxiella burnetii* in *Camelus dromedarius* camels and *Hyalomma dromedarii* ticks.

**Country**	**No. of camels tested**	**% of camels with *C. burnetii* Ig**	**No of ticks tested**	**% of ticks with *C. burnetii* Ig**	**Diagnostics tests**	**Related human outbreak (or not)**	**References**
Algeria	184	71.2% (131/184)	0	ND	**Serological test:** *C. burnetii* Indirect Multi-species ELISA Kits (ID Screen®)	ND	(378)
Canary Island	100	19% (19/100) (0% by PCR assay)	0	ND	**1. Serological test:** LSIVET™/ruminant milk/serum Q-fever **2. Molecular techniques**: Conventional PCR	ND	(380)
Chad	500	4.8% (24/500), up to 28.6%	0	ND	ND	ND	(381)
Chad	613	80% (490/613)	0	ND	**Serological test**: *C. burnetii* Indirect Multi-species ELISA Kits	Coxiellosis may be responsible for several undefined cases of fever	(105)
Egypt	0	ND	(batch of 54 ticks)	ND	ND	ND, not a notifiable disease	(382)
Egypt	200	66% (132/200)	0	ND	**Serological test**: 1. Conventional enzyme immunoassays (EIAs) 2. Competitive enzyme immunoassay (CEIA)	ND, not a notifiable disease	(373)
Egypt	332	13.3% (4/332)	0	ND	Conventional IFA antibodies	ND, not a notifiable disease	(171)
Egypt^b^	528	40.7% (215/528)	0	ND	**Serological test:** CHEKIT Q fever Antibody ELISA Test Kit	ND, not a notifiable disease	(235)
Egypt	113	46.0% (52/113)	177	5.6% (10/177)	Molecular techniques: PCR	ND, not enough available data	(383)
Egypt^c^	112	4.5% (5/112) (16.9% by PCR assay)	0	ND	**1. Serological test**: *C. burnetii* ELISA kit (GSCIENCE, USA) **2. Molecular techniques**: -Real-time PCR-Conventional PCR	ND, not enough available data	(374)
India	ND	17.3%	0	ND	ND	ND	(384)
India	ND	6.6%−7.7%	0	ND		ND	(385)
Iran^d^	167	28.7% (48/167)	0	ND	**Serological test:** CHEKIT-Q fever ELISA kit	ND	(370, 375)
Kenya	ND	20.0%	0	ND	ND	ND	(386)
Kenya	334	18.6% (62/334)	0	ND	**Serological test**: The CHEKIT Q fever by IDEXX *C. burnetii* antibody	ND	(371)
Kenya	312	19.9% (62/312)	0	ND	**Serological test**: CHECKIT Q Fever Antibody ELISA Test Kit	ND	(379)
Nigeria	386	11.4% (44/386)	0	ND	ND	ND	(387)
Saudi Arabia	460	62% (285/460)	0	ND	**Serological test**: CHEKIT-Q fever enzyme immunoassay	ND	(376)
Saudi Arabia^e^	489	51.6% (252/489)	0	ND	**1. Serological test**: CHEKIT-Q fever enzyme immunoassay **2. Molecular test**: Conventional PCR	ND	(372, 377)
Sudan	ND	12.8%	0	ND	ND	ND	(388)
Sudan	ND	14.5%	0	ND	ND	ND	(389)
Sudan	76	64.3% (49/76)	0	ND	**Serological test**: Commercial Q fever antibody indirect ELISA test kits	ND	(214)
Tunisia	ND	15.8%	0	ND	ND	ND	(390)
Tunisia	534	44.0% (235/534)	0	ND	**Serological test**: Commercial Q fever antibody indirect ELISA test kits	ND	(391)
Tunisia	412	0^f^	327^g^	3.6% (12/327)	**Molecular test:** Conventional PCR	ND	(392)

*ND, not determined*.

*^a^In this study, the seroprevalence was 4.8%, but it should be noted that 16 of the 24 positive animals were from a single herd containing 56 heads of camel, which correspond to a seroprevalence of 28.6% in this herd*.

b *In this study, most of the camel samples tested were collected from animals imported from Sudan, and the seroprevalence in camels from the Aswan governorate of Egypt bordering Sudan was 67.5%. Hyalomma dromedarii ticks are commonly found in Egypt (393)*.

c *In this study, the camel samples were tested for anti–C. burnetii Ig and 4.5% of camels were found positive. Additional evaluation by standard PCR using the superoxide dismutase enzyme of C. burnetii indicated that 16.9% (19/112) of camels were found positive*.

d *In this study, the camel samples were collected from animals living in 11 different counties, and the seropositivity ranged from 0 to 63.6%, depending the counties of origin of the camels; Hyalomma dromedarii ticks are commonly found in Iran (394, 395)*.

e *The highest seroprevalence was recorded in Magahim camels; some harbored the camel tick Hyalomma dromedarii*.

f *C. burnetii seroprevalence estimated by PCR was not detected in any of the 412 samples collected on dromedary camels in Tunisia, despite that their ticks were found positive by the same assay, and seroprevalence previously determined by anti–C. burnetii Ig was estimated between 15 and 44% in Tunisia. The authors argued that lack of detection in camel samples was probably due to the low load of bacteria in animal blood. They admit this result is different from those reported in Egypt, Saudi Arabia, and Iran, which demonstrated the direct identification of C. burnetii by PCR in camel blood*.

g *C. burnetii was estimated by PCR and found in 3.6% of 327 ticks. Hyalomma impeltatum was the most infected ticks species, 5.7% (9/158), followed by Hyalomma dromedarii, 1.9% (3/160). The frequency of tick infestation was higher when collected on camels located in the governorate of Gabes*.

It could also be interesting to investigate the role of the camel hump adipocytes in the long-term storage of *C. burnetii*. In a murine model, it was demonstrated that once *C. burnetii* has gained the host bloodstream, during the first week of infection it penetrates different organs, and bacteria can be found in spleen, liver, epididymis, prostate, and semen. At 3 weeks, degenerative changes in capillary blood vessels and the surrounding tissues of the adipose envelope of the epididymis are concomitant to the circulation of infected macrophages, and bacteria shed to semen can be transmitted from male to female by sexual intercourse (404). At 4 months postinfection, *C. burnetii* was detected in abdominal, inguinal, and dorsal adipose tissues, whereas no bacteria were detected in blood, liver, lung, and spleen, and the transfer of adipose tissue from convalescent mice to naive immunodeficient mice resulted in the infection of the recipient host (405). Altogether these results acquired in other models than camels indicate that adipose tissues may be the reservoir in which *C. burnetii* persists for prolonged periods after the end of clinical symptoms. Although infection by *C. burnetii* of camel hump adipocytes has not been evaluated so far, the elevated concentration of adipocytes in camel hump could provide *C. burnetii* with an ideal long-term storage site unique among the ruminants (406). Moreover, when food is scarce, *C. burnetii* could be released from hump adipocytes during lipolysis. During dehydration and underfeeding periods, camels mobilize their hump adipose tissue accumulated during overfeeding periods to compensate for the deficit (406). In the pastoral communities, the close physical contacts with dromedary camels create the conditions for the transfer to the man zoonotic diseases. A meta-analysis that searched in nine databases, the 929 unique articles regarding *C. burnetii* epidemiology in Africa concluded that close contact with camels was associated with increased seroprevalence in humans (95).

## Domestication and Breeding of the Dromedary Camel (*Camelus dromedarius*): A Socioeconomic Role In the Life of Millions Of People

**Figure 5:**
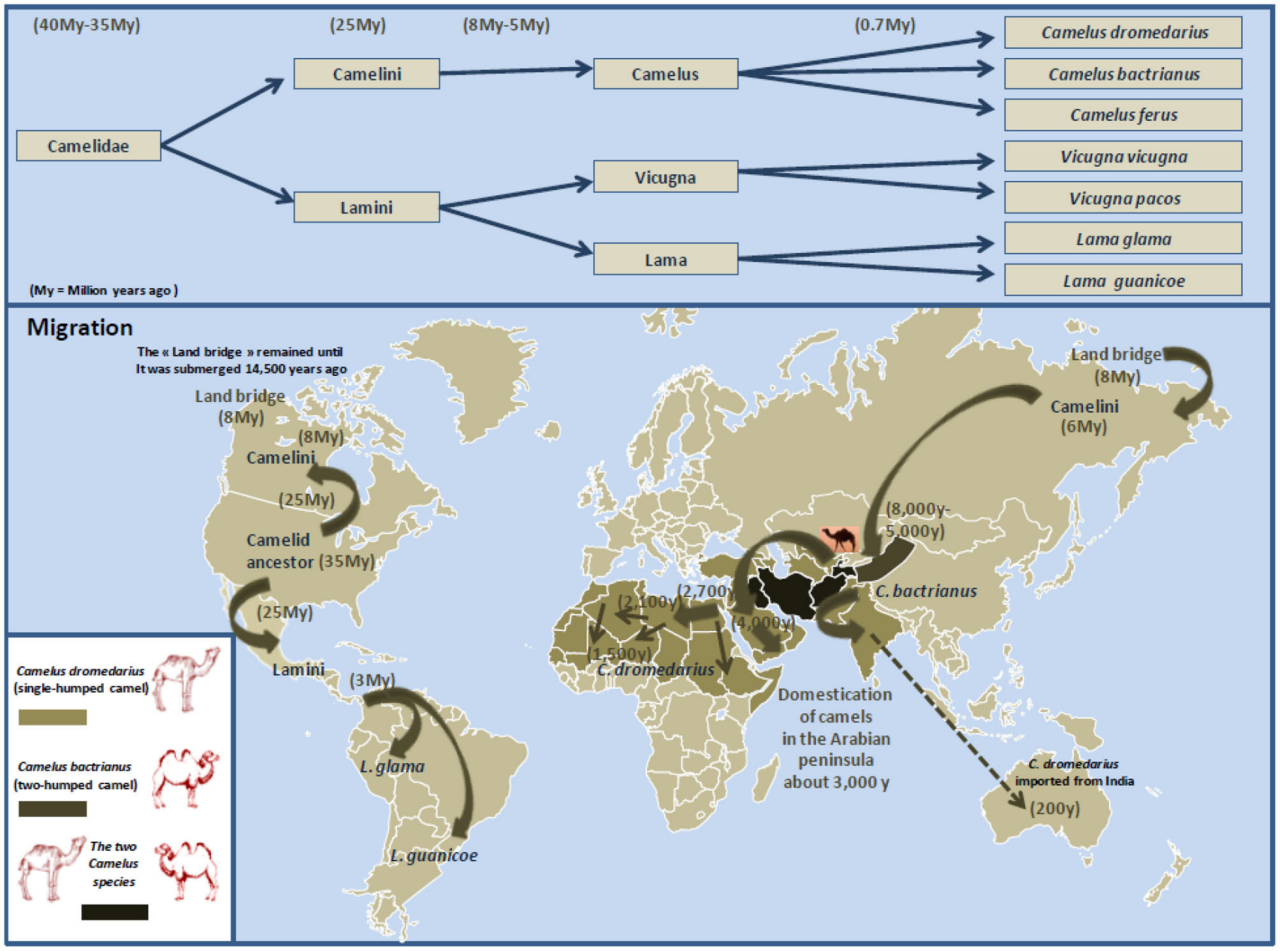
Schematic representation of Camelidae evolution, migration, and domestication. The earliest known Camelidae, named *Protylopus* and *Poebrotherium*, appeared roughly 40 million years ago in the North American. During the transition from the Eocene to the Oligocene geological period (about 34 million years ago), the climate in North America is expected to have changed for cooler and drier, and Camelidae began to genetically diverge (407). This was supported by the discovery of the fossil of *Paracamelus* in Canada in 1913 where this ancestor of *Camelus bactrianus* and *Camelus dromedarius* was expected to have inhabited there about 3.5 million years ago when a warmer climate allowed forests to spread near the Arctic Circle (408). About 6 to 8 million years ago, Camelini gradually moved across the land that connected North America to Asia (this land bridge appeared some 8 million years ago, and it remained practicable until it was submerged about 14,500 years ago). According to Wu et al. (409), during the species evolution, they diverged into (i) large Camelidae (Camelini), which lived in North America and next moved westward across the land that connected North America with Asia, then Middle East and North Africa; and (ii) small Camelidae (Lamini), which dispersed South (currently South America). Subsequently about 5 to 8 million years ago, Camelini further evolved into *Camelus*, which include two species: *Camelus bactrianus* (the two-humped camel; weight: 600–1,000 kg; size 1.6–1.8 m) and *Camelus dromedarius* (the single-humped camel; weight: 400–600 kg; size 1.6–2.0 m). Lamini subdivided into two genera: *Lama* and *Vicugna*. The earliest evidence for the dromedary domestication is dated about 3,000 years ago near Abu Dhabi on the Arabian Gulf. Northern Arabian tribes began to use dromedary camels as riding animals (410). Dromedary camels were progressively domesticated in North Africa. Gift of camels was a source of camel spread around the Mediterranean. Currently, there are 33 million of domestic *Camelus dromedarius* living in semiarid and arid regions of Africa and the Middle East, 3 million of domestic *Camelus bactrianus* that live from the cold steppes of Central Asia to the border of Manchuria in China, and a small population (1,000 camels) of *Camelus ferus*, the Wild Bactrian, which survives in the Northwest China and the Gobi Desert of Mongolia (*Camelus ferus* diverged from *Camelus bactrianus* about 0.7 million years ago) (411). Dromedary camels from India were also introduced in central Australia. Females are only able to conceive from 3 years old and can live up to 30 to 40 years old.

The large camelids include two domestic species: *Camelus bactrianus* (the two-humped camel) and *Camelus dromedarius* (the single-humped camel) (Figure 5). Regarding the bactrian camel, a strain adapted to cold winters that inhabit mainly the mountains of central Asia, historians reported that the camel production was already recommended in the pre-Islamic sacred religious books (412). The dromedary camel, *C. dromedarius*, nicknamed “desert vessel,” was domesticated in the Arabian Peninsula around the 1st millennium and the second century bc (413–418). It is usually considered that the dromedary camel domestication appears late compared to other ruminants because it took place about 8,000 years after that of sheep and 6,000 years after that of cattle (419, 420). The use of the dromedary camel gradually developed with caravan trade of spices in the Arabian Peninsula and Mediterranean cities markets.

Dromedary camel domestication was crucial for livelihood of pastoral communities in which camels are kept for multiple uses including transport of people (camels can travel several hours per day at a speed of 15–20 Km/h), transport of loads (they can carry between 150 and 250 kg), the maintenance of an agricultural activity around oasis, the control of desertification and rational management of water resources, milk production and consumption, source of meat, and traditional medicine (421–423). Camels feed on herbaceous plants, shrubs, shoots, cacti, and date stones and can spend months in semiarid regions without drinking (424, 425). During millennia, camels were reared according to three breeding systems: sedentary, nomadic, and transhumant. Given the ecological zone in which they live, the last two systems are the most frequent, with a predominance of the transhumant mode (426–428). In most areas, dromedary camels are multipurpose animals with the females used primarily as milk producers and the males for transport or draft. The usual selection criteria of dromedary camels were color, morphometric characteristics, milk production, and endurance. For example, the Guerzni type is a pack camel maintained by nomads; the Marmouri type is a dromedary camel used for riding, whereas the Malhah- and Wadhah-type breeds were selected for high milk production (429, 430).

Economically, dromedary camel exploitation appear problematic because of slow reproductive cycle (13 months of pregnancy) and high mortality of young (431, 432). Reproductive losses in camel herds are due to infertility (uterine infection), pregnancy loss (infectious pathogen–induced abortions), mastitis (female udder infections), and neonatal diseases (433). A large investigation (11,200 camels from different herds) in Ethiopia regarding the major constraints to camel production emphasized widespread diseases, lack of attention to camels, lack of experience and knowledge, inadequate veterinary service, lack of attention by the government, poor infrastructures, and feed shortage. Yet, camel production remains attractive for low-income people, and renewed interest for camel breeding was observed in the Maghreb (e.g., Morocco) because of the increasing food needs for urbanized population. For meat consumption, at 3 years old, the weight of dromedary camels can reach about 500 kg (251). Regarding milk, the production of dromedary camel milk varies within camel breeds (434). The Hoor Somalian breed can produce 8 L of milk per day for 8 to 16 months, whereas the Eydimmo breed can produce 4 L of milk for 6 to 12 months (435). Camel milk is considered the closest to human mother milk, highly nutritious and with high minerals and low sugar and cholesterol (436). More recently, a fourth dromedary camel breeding mode has been developed that is camel breeding farms.

**Figure 6:**
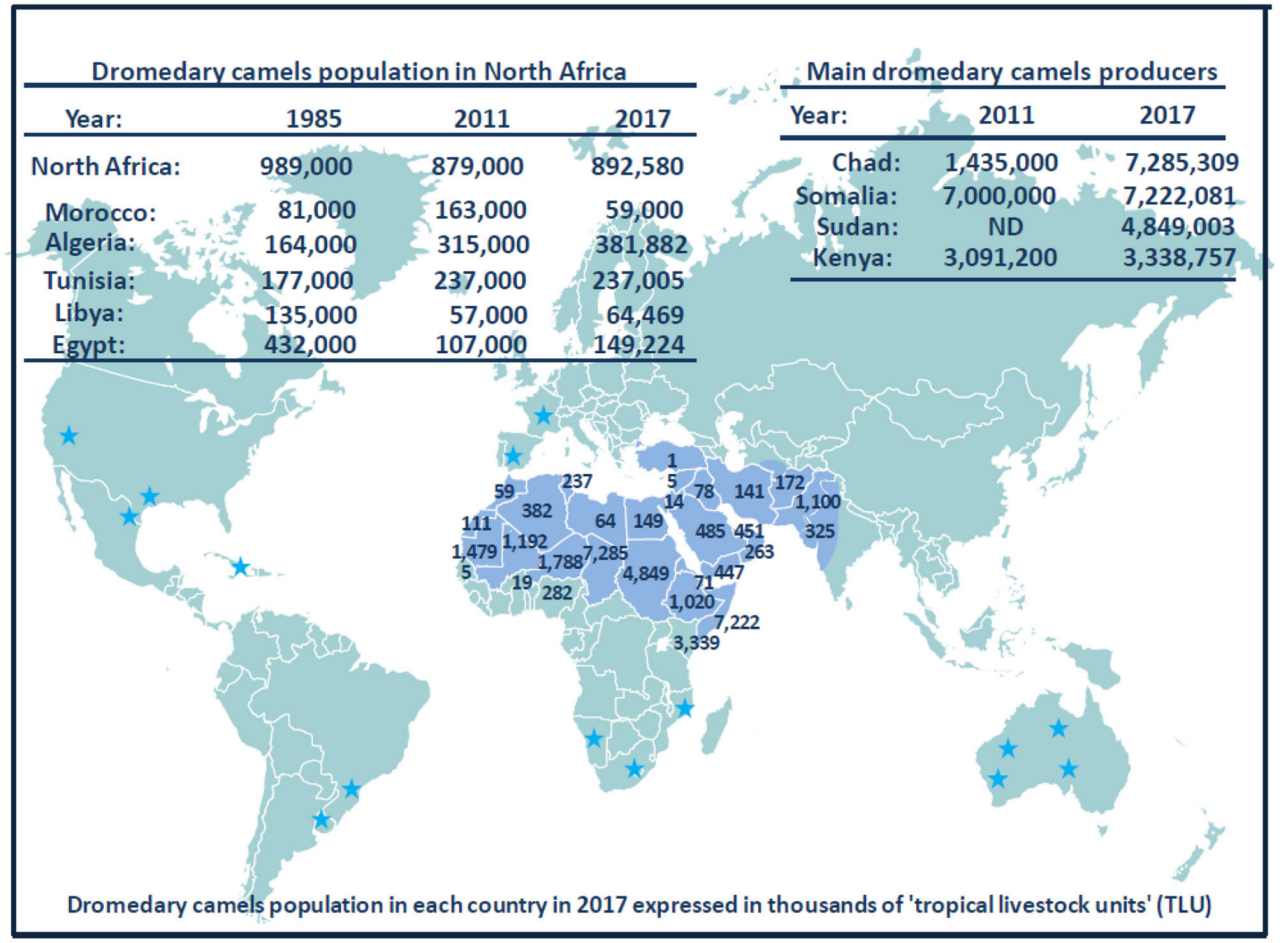
Schematic representation of dromedary camel distribution in the world. The Arabic generic term commonly used for camel is “ibl.” Male camel between 6 and 20 years old are named “jamal” (also the Arabic word for *beauty*). Currently, there are ~33 million dromedary camels (or humpback camels) worldwide, with highest numbers (77%) in Africa and the Middle East. The geographical location of the dromedary is in the belt of the tropical and subtropical dry zones of Africa, but its presence extends to Western Asia and Northwest India (blue area on the map). Dromedary camels are found in 35 native countries ranging from Senegal to India and from Kenya to Turkey. The number on the map show the population of dromedary camels in each country in 2017 expressed in thousands of “tropical livestock units” (TLUs), according to FAO (369). For example, the largest dromedary camel populations are found in Chad (7,285,309 camel heads) and Somalia (7,222,081 camel heads), followed by Sudan (4,849,003 camel heads) and Kenya (3,338,757 camel heads). Regarding the countries of the Southern coast of the Mediterranean Sea, the current livestock situation is as follows: 59,000 camels for Morocco; 381,882 camels for Algeria; 237,005 camels for Tunisia; 64,469 camels for Libya; 149,224 camels for Egypt; 5,530 camels for Israel; 66,390 camels for Syria; and 14,322 camels for Jordania. A massive dromedary camel (300,000 now-feral dromedary camels) implantation was made in the last century in Australia from camels imported from India (not shown); very specific introductions were also made in the United States, Central America, South Africa, and Europe; they are indicated by blue stars (437). Another member of the Camelidae family, the *Camelus bactrianus* (or two-humped camels) with a distinguished geographical distribution is present from the cold deserts of Central Asia to the border of Manchuria in China (see Figure 3). Both *C. bactrianus* and *Camelus dromedary* species can cohabit in a few places such as western Asia.

Currently, the population of dromedary camels is ~33 million heads (Figure 6), with highest numbers in Africa and the Middle East. The numbers of dromedary camels from one country to another have been very variable over the last 50 years. In the countries of the Southern shore of the Mediterranean, the population of dromedary camels had drastically declined in Palestine, Syria, Lebanon, and Turkey between the 1960s and 1990s, rising from 89,000 to 10,550, and then returning to growth with 62,000 heads in 2011; most of them (about 54,000) breed in Syria. In 2017, the Syrian livestock of camels was 66,390 heads. For the countries of North Africa (Morocco, Algeria, Tunisia, Libya, Egypt), the total population decreased from 1,031,000 heads in the 1960s to 879,000 in 2011 with 163,000 heads in Morocco; 315,000 in Algeria; 237,000 in Tunisia; 57,000 in Libya; and 107,000 in Egypt (369). The density of dromedary camels per inhabitant has been estimated at one dromedary camel per 45 humans in Tunisia, one per 98 in Libya, one per 119 in Algeria, one per 200 in Morocco, and one per 792 in Egypt, but these values calculated on the global populations of dromedary camels and humans do not reflect the regional discrepancies (438). For example, in Morocco, 58% of dromedary camels are found in the Southern Saharan region and 26% in the East-West band from Ouarzazate to Figuig passing by Rachidia (430). It should be noted that between 2011 and 2017, Tunisia has stabilized its livestock, whereas the population of dromedary camels increased in Algeria and Egypt and decreased in Morocco and Libya. In the country producing the largest livestock in Africa, Somalia kept almost stable livestock with 6,411,000 camels in 1985, 7,000,000 in 2011, and 7,222,081 in 2017, whereas Chad showed a marked increase in livestock with 481,060 in 1985, 1,435,000 in 2011, and 7,285,309 in 2017. The highest density of camels by land area or human population in the Arabian Peninsula is found in UAE and Qatar (439), with 451,463 camel heads and 9.4 million inhabitants (one camel per 21 inhabitants) in UAE, and 40,843 camel heads for 2.6 million inhabitants (one camel per 63 inhabitants) in Qatar in 2017, respectively. In the early 2000s, the relative importance of camels with respect to the total animal biomass was 6.2% in Africa, 0.7% in Asia, and 15.1% in the Arab countries, respectively. Moreover, the total world meat and milk productions from camels were about 376,000 tons/year, and 5,100,000 tons/year (440). In the Arab countries, the animal biomass was mainly composed of cattle (54.8%), followed by sheep (13.6%), camels (10.1%), goats (8.8%), buffalo (8.0%), and equine (4.6%) (440). In countries such as Sudan, Niger, Chad, and Tunisia, camel breeding represents a significant part of the agricultural economics, whereas it is of primary importance in the economy of Somalia, Mauritania, and Djibouti (427). As shown in Table 5, in 2017, the production of camel milk in Somalia was 953,673 tons, whereas it was 26,470 tons for Morocco, Algeria, Tunisia, and Libya altogether.

**Table 5:** Production of whole fresh camel milk and indigenous camel meat in North Africa compared to other countries of Africa and Arabic peninsula.

	**2011**		**2017**	
**Camel production (Tons)**	**Milk^a^**	**Meat^b^**	**Milk**	**Meat**
**Country**				
Morocco	8,374	4,296	8,374	3,000
Algeria	13,500	5,190	14,004	5,948
Tunisia	1,200	1,435	1,092	ND
Libya	2,500	5,105	3,000	ND
Egypt	ND	28,871	ND	ND
Chad	18,004	1,242	64,634	ND
Somalia	1,080,000	62,644	953,673	ND
Sudan	ND	ND	60,897	ND
Kenya	890,276	64,500	876,224	ND
Djibouti	6,456	12,357	6,043	ND
Saudi Arabia	98,000	22,112	134,266	ND
UAE	45,535	27,296	54,024	ND
Yemen	12,851	2,720	13,431	ND
Qatar	5,277	ND	8,590	ND

a *Tons of whole fresh camel milk according to FAO (369)*.

b *Tons of indigenous camel meat according to FAO (369). Many countries do not only consume locally produced animals but also import dromedary camels raised in other countries*.

After a decrease of the dromedary camel populations in several countries, the recovery can be associated with the increasing demand for milk and camel meat that parallel the increase of human population (441). Unlike camel meat production whose market price is lower than that of sheep and cattle meat, milk production remains poorly valued, and its price is higher than that of cow's milk (428). In the case of Tunisia, the camel red meat production increased from 2,150 tons in 1997 to 3,500 tons in 2003 (439). Regarding North Africa (Morocco, Algeria, Tunisia, Libya, Egypt), the dromedary camel production systems are characterized by major differences in the sizes of herds ranging from a few heads in the agropastoral systems to thousands of heads. Some highly intensive farms are currently emerging all over the Arabian Peninsula both for milk production (including pasteurized milk) and meat production (feed-lot farms of young male camels, named *hachi*) (274). The Maghreb countries and Egypt favor import (from Sudan, Ethiopia, and Chad), rather than breeding of camels. Libya imports camels mainly from Chad and Niger for food. Only Tunisia is self-sufficient. Because of desertification, camels may become an interesting issue to replace cattle as a source of milk and meat in the newly desertified areas of the world. Indeed, adaptation of camels to desert ecosystems has attracted the attention of international organizations including the International Fund for Agricultural Development, the Islamic Development Bank, which together with a funding agency from the French Government have established the Camel Applied Research and Development Network (CARDN) in 1991. These funding agencies have next contracted with the Arab Center for the Studies of Arid Zones and Dry Lands, which started to operate as the executing agency of the consortium since 1996 to develop camel husbandry. CARDN supports laboratories, units for artificial insemination and embryo transfer, and mobile veterinary units. Several Southern countries are at a socioeconomic crossroads, which means choosing to modernize the sector in order to improve the productive performances of dromedary camels. Studies on production and marketing of camels were conducted in Tunisia, Egypt, Sudan, Pakistan, and Mauritania. In 2017, date of the latest statistics available according to the FAO, the population of dromedary camels in North Africa was 891,000 heads in North Africa, with 59,000 heads in Morocco, 382,000 in Algeria, 237,000 in Tunisia, 64,000 in Libya, and 149,000 in Egypt (369).

## Besides Red Meat and Milk, Dromedary Camels are A Source of Income in Tourism and Animal Racing

Besides food, camels play a role in local tradition and economy. The hides and skins sector, long neglected, is improving rapidly. Tunisia and Egypt develop good practices for killing the animals and better quality of tannery treatment. Tunisia has thus created a technical center leather and footwear, which is interested in dromedary product valorization. It is especially in the field of recreation and tourism that the dromedary camels know a continued interest, either to animate meharias in the desert (although this activity has decreased in some countries for security reasons), or as part of scenery of tourist places (e.g., camel rides at the foot of the pyramids in Egypt, at Djerba in Tunisia, or Essaouira in Morocco). The riding of the dromedary camels as saddle animal is regularly practiced in most countries of the Maghreb, particularly among the Saharawis (Morocco) or Tuareg (Algeria, Libya) populations. Dromedary camels are also attracting tourists around races that are very popular in the countries of the Arabian Golf and North Africa (432, 442, 443). Over many years, typical racing dromedary camels (slim, lightweight, high-speed) were selected to confer highest sports performance (Table 6). The racing stables are maintained with great care, selected feeding, and training of animals. In North Africa, racing of dromedary camels is the occasion of festivities like the Douz marathon (Tunisia), the festival of Marrakech (Morocco), or the fantasia of Ouargla (Algeria). In the Arabian Peninsula, camel shows called Mazayin al-Ibl (“best of camels”) are held annually with the 100-camel herds competition day and the camel beauty contest. One of the largest camel shows (about 160,000 camels) is usually held in Um al-Rughaiba (300 km from Riyadh in Saudi Arabia), where thousands of people come to attend the show (459). During thousands of years, Arab Bedouins have bred camels for speed and endurance, whereas camel racing became a professional sport in the UAE only after discovery of oil. Today, camel racing is considered a strategy to reinforce national identity by preserving the ancestral heritage in a modern country (460). Dromedary camels in the UAE are mainly grouped into three breeds (Al-Arabiat; Al-Kazmiat; racing camels); the government imported well-known racing camels from different countries, and they were used for endogenous breeds leading to new racing breeds including Sokan, Hamlol, Msehan, and Al-Thenian (444). Several Omani camels have been selected for racing such as Al-Azkiyah and Al-Bahree famous for short-distance racing or Kudsha and Arjaa famous for long-distance racing. In Saudi Arabia, there are 4 main camel breeds (Al-Majahem; Al-Makater; Lorak; racing camel breeding of Al-Omaniat, Al-Hurah, and Al-Sodaniat) (444). In a past period now over, the UAE and other Gulf states involved child jockeys in camel racing, drawing lawsuits from human rights groups (461). This led to a change in practices and the founding of the Camel Racing Association in 1992 and Camel Racing Federation in 2003. The practice of child jockeys was banned, and since then, camels are spurred on by small robots jockeys. There are several racetracks across the country with spacious and well-kept stadiums for viewers. The Abu Dhabi Authority of Culture and Heritage annually organizes a famous camel international festival in April. Another major racing competition is held in February in the Janadrriyah suburb of Riyadh (459). The King Abdulaziz Camel Festival (28 day celebration) attracts more than 300,000 people, almost 2,000 owners, and 40,000 camels. The winner of a beauty contest can get a prize of several 100 thousands of US dollars. In Saudi Arabia, $57 million are distributed annually in Camel Festivals. Several other international camel festivals are held annually in Oman, Qatar, and Kuwait.

**Table 6:** Examples of the most popular camel breeds in the North Africa and Arabian Peninsula.

**Country**	**Most common use**	**Breed name (coat color)^a^**
Morocco^b^	Dairy camels	Ouled Sidi Cheikh (dark); Marmouri (dark); Guerzeni (dark)
	Multipurpose vocation camels	Khouari (light brown, blondish)
Algeria^c^		
	Racing camels	Azawad (light/white); Regbi (light); Targui (white/clear)
	Dairy camels	Ouled Sidi Cheikh (dark); Rguibi (clear/white); Barbari (various coat color)
	Multipurpose vocation camels	Hamra (reddish brown)
Tunisia^b^	Multipurpose vocation camels	Maghrebi (mainly reddish, various coat color)
Libya^b^	Multipurpose vocation camels	Sirtawi; Alarabia (white, light brown, gray); Almaharee (blue, yellow); Altebestee (yellow, sand color), alsertawiya (light brown, dark brown, or blue)
Egypt^d^	Racing camels	Somali (off-white); Sudani (various coat color)
	Dairy camels	Maghrabi (various coat color)
	Multipurpose vocation camels	Mowalled (various coat color)
	Transportation, agriculture	Falhi (various coat color)
Saudi Arabia^e^	Racing camels	Asail (yellow to brown); Shageh (gray); Zargeh (blue-gray)
	Dairy camels	Harnor (brown); Majaheem (black); Safrah (dark brown); Shaele (gray); Sofor (darkbrown); Waddah (white)
	Multipurpose vocation camels	Aouadi (reddish to white); Awrk (white); Hadhana (light brown); Maghateer (white); Saheli (reddish)
United Arab		
Emirate^f^	Racing camels	Samha (brownish-red); Farha (red, blond or yellow); Al-Bahree (reddish to yellowish); Al-Azbah (blondish); Kudsha (reddish-blondish); Sadoorah (light red)
	Dairy	Arjaa (yellowish-blondish); Shahbar (reddish to blondish)
	Multipurpose vocation camels	Al-Azkiyah (light yellowish); Al-Kawara (reddish to yellowish); Dhibian (reddish); Zabeia (blondish); Al-Derehiah (yellowish-blondish)

*Adapted from Kadim and Mahgoub (444); FAO (445); and Ali Fouad et al. (446)*.

a *Among breeders, the genetics of camels is based on phenotypic and sociogeographical criteria (in relation with the ethnic groups of breeders in the different regions). The camel populations exhibit variability in certain phenotypic and morphological traits such as size (shoulder height for dromedary camels: 1.6–2 m; shoulder height for bactrian camels: 1.6–1.8 m), color of the dress and fineness of the coat (white, gray, yellow, brown, reddish, dark, black), and hair structure and adaptive traits such as hardiness (disease resistance and drought tolerance) animals. There are 90 recognized breeds of dromedary camels and 14 bactrian breeds. For more details regarding the popular camel breeds for Somalia, India, Pakistan, China, and Mongolia, see reference Ali Fouad et al. (446)*.

b *Wardeh (447); Guerouali and Acharbane (430); Bakory et al. (448); FAO (445)*.

c *Aissa (449); Amine et al. (450); Cherifi et al. (451); Ali et al. (452)*.

d *Mukasa-Mugerwa et al. (453); Ramadan and Inoue-Murayama (454); Ali et al. (452)*.

e *Abdallah and Faye (455); Massad (456); Abdelrahman et al. (457); Al-Atiyat et al. (458); Ali et al. (452)*.

f *Kadim and Mahgoub (444)*.

Although racing camels receive a lot of attention at the sanitary level, outbreaks of bronchopneumonia and gastroenteritis sometimes affect racing camels (311, 462). Yet there has been so far no report of *C. burnetii* outbreak in racing camels. Knowledge about the genetic characteristic and diversity of camels is improving (463–465). The size of camel genome is roughly 2 to 2.4 GB, encoding for more than 20,000 genes (452, 466). Growing interest in racing camels has led to set up research centers (e.g., Camel Research Center at King Faisal University in Hofuf, Saudi Arabia, and Camel reproductive center in Dubai) aimed at improving breeding stock. In 2009, in Dubai, the world's first successful cloning of a *C. dromedarius* was reported (467). More recently, the same research center reported the first cloning of a *C. bactrianus* (468).

## What Threat Does *C. burnetii* Represent for Humans and Camel Breeding?

Q fever is transmitted to humans through inhalation or ingestion of infected animal products such as meat, milk, or cheese. Camel milk is a major component of the diet in many pastoralist societies. When nomads move in search of pasture, they can live for up to a month in the desert on nothing but dromedary camel milk. Daily female camel milk production ranges from 2 to 6 L under desert conditions and up to 20 L under a more favorable environment (469). Most camel milk is drunk fresh, which may be a source of infection if the animal excretes *C. burnetii* in milk. With increasing urbanization, it has gained a wider market, and commercialization and consumption of camel milk are on the rise (470). Every year, 5.4 million tons of camel milk are produced (369). Although not extensively investigated for camel, the rate of excretion of *C. burnetii* in milk would be expected to be low, except in the early days after parturition (471).

There is also an increase of camel meat consumption, which is parallel to the urban development (441). For example, there has been a radical change in dromedary camel farming practices in the Arabian Peninsula since the 1960s, with an intensification of the production around cities. The annual camel meat consumption is estimated to be 21,500 tons in Saudi Arabia, a country where 33 million people are living (369). This change in methods of breeding camels might increase the frequency of zoonotic infections from camels to humans. It seems reasonable to assume that the sensitivity of public health surveillance to detect infectious microorganism in camels and to investigate the source of sporadic human cases of infectious diseases is higher in Saudi Arabia and then developing countries of East Africa. However, camel meat consumption is also very high in the Africa countries (e.g., 6,000 tons in Chad, 3,000 tons in Niger) (369). In Djibouti, where a population of about 1 million people is living and with a livestock of 70,965 camels (one camel per 14 humans), 300 tons of camel meat are eaten annually. In several North African countries, there is a long supply circuit of camel meat with several intermediary operators who, for example, carry herds from the El-Obeid region in Sudan to Aswan in Egypt where the dromedary camels are killed and dispatched on markets. The cross-border trade of camels from Sahelian countries to North Africa could represent a sanitary risk since the percent of Sahelian animals exposed to, or infected by, *C. burnetii* seems to be very high. Disease transmission associated with cross-border transport of dromedary camels was previously documented for RFV and pestis (472, 473).

In the past decade, at-risk populations for *C. burnetii* infection were limited to pastoral communities, farmers, slaughterhouse and tannery workers, veterinarians, and raw milk drinkers. With the increasing demand of milk and camel meat in urban areas, there is a potential threat for millions of people (474). Because camels suffer from lack of attention in several countries, the control of *C. burnetii* within livestock is severely hampered. Insufficient serological surveillance and uncontrolled trade of infected animals may therefore have direct consequence on the flock sanitary evolution. Effort should be made to increase awareness of Q fever in public, veterinary health authorities, and decision makers. Human is not the sole species at risk during meeting with infected camel. The bacteria can spread intraspecies in dromedary camel flocks, and interspecies transmission to other cattle remains possible. This could possibly impact the economy of affected countries and also their food reserves.

## Fighting the *C. burnetii* Threat in Dromedary Camel Herds and Farms

To fight against *C. burnetii* transmission (camel-to-humans, camel-to-camel, camel-to-other livestock), global hygiene procedures should be introduced (rational vaccination schemes, antibiotics, disinfectants, hygiene procedure to handle the products of abortion). Appropriated management of the risk requires clear information of camel keepers/owners, regular investigations of the animal sanitary statute by veterinary (regular serological tests would improve surveillance) crosstalk and collaboration between the veterinary and medical sectors, the establishment of health guidelines in all countries concerned, and intergovernmental cooperation between trading countries. Sometimes, very simple procedures may also improve the health status of herds. For example, to control mastitis in camel, it is a good practice to remove ticks, even when the animal is not lactating (475).

Regarding the general hygiene measures, the farmer must keep the premises clean. The professionals must wear a mask and gloves in areas expected to be possibly contaminated, remove afterbirth and birth fluids, and disinfect areas where camels have given birth and material in contact with camels. Immediate reporting of outbreaks is required to quickly start investigations of camel farms, other livestock, and domestic animals. Killing infected animals remains a possible strategy in extreme uncontrolled situations.

In an epidemic case, it should be recommended to pregnant women to avoid participating in farming activities, and vaccination of professionals should be considered. A vaccine against human Q fever was developed using the formalin-inactivated *C. burnetii* Henzerling strain phase I (Q-Vax®, Commonwealth Serum Laboratories, Parkville, Victoria), but it is distributed only in Australia (88, 90, 476–478). The availability of a human vaccine for at-risk professionals would be of benefit to prevent human outbreaks. In case of febrile illness following contact with ruminants, the diagnosis of human Q fever is established by serology and bacterial identification (8). Molecular techniques have an added value to the diagnostic of acute Q fever and for the clinical follow-up of infected people. Infected people should be treated with doxycycline 100 mg twice daily for at least 2 weeks. In case of gastric intolerance to doxycycline or in the case of meningoencephalitis, fluoroquinolones (ofloxacin 200 mg three times a day or pefloxacin 400 mg twice daily) are preferred (18, 479–481).

In farms, the prevention of *C. burnetii* shedding by infected animals (sheep, goats) is possible through vaccination of livestock with a phase I vaccine (482). Formaldehyde-inactivated whole *C. burnetii* made with phase I antigens confers greater protection than those made with phase II antigens (483, 484). To counteract the undesirable effects (induration, abscesses) of formaldehyde inactivation, chloroform–methanol vaccines were proposed (485, 486). A trichloroacetic acid–treated *C. burnetii* phase I vaccine is used in Slovakia (487). During the 2007–2010 Q fever outbreak in the Netherlands, vaccination of livestock was used to reduce the transmission of *C. burnetii* to humans. In France, an inactivated *C. burnetii* phase I vaccine (Coxevac®) was found to efficiently protect goats against abortion. However, vaccination did not clear infection in previously infected goats and cattle (488–490). More recently, a vaccination of red deer with Coxevac® was found to reduce *C. burnetii* shedding in feces, but not yet in vaginal secretions and milk (491). *C. burnetii* phase II vaccines protective for small ruminants have been developed in France (Chlamyvax-FQ® from Merial and Abortstop® from Rhône-Poulenc Rorer) (492). In already infected animals, these vaccines seem to only reduce *C. burnetii* shedding in feces. So far, there are no data available regarding the vaccination of camels against *C. burnetii*.

Currently, it is of major importance to monitor both the camel herds and camel farms. The search for anti–*C. burnetii* antibodies must be carried out on the serum in an abortive context. This low-cost method is not the most reliable, with a significant percentage of false-negative results but useful for rapid milk screening. Detection can also be performed on tissue samples using Gimenez staining. The sample must be made as sterile as possible and be quickly transported to the laboratory (within 48 h at 4°C; otherwise, it must be frozen). Because of their higher sensitivity and specificity, immunofluorescence or immunoperoxidase immunodetection assays should be preferred to microscopic identification. The two most reliable methods are enzyme-linked immunosorbent assay and PCR. PCR diagnosis using specific oligonucleotide probes is probably the fastest, most sensitive, and feasible method. Yet, it is still too expensive to use it routinely in monitoring of camel herds. A less expensive strategy could consist in testing pool of samples from 10 animals and return to individual tests only in case of a positive result. Finally, the culture of the bacteria on agar followed by *C. burnetii* identification by matrix-assisted laser desorption/ionization–time-of-flight or genomic sequencing is rarely used in veterinary medicine, but it brings irrefutable proof of the infection and can allow the comparison of strains isolated in camels, humans, and the ecosystem to identify the reservoir of bacteria.

**Figure 7:**
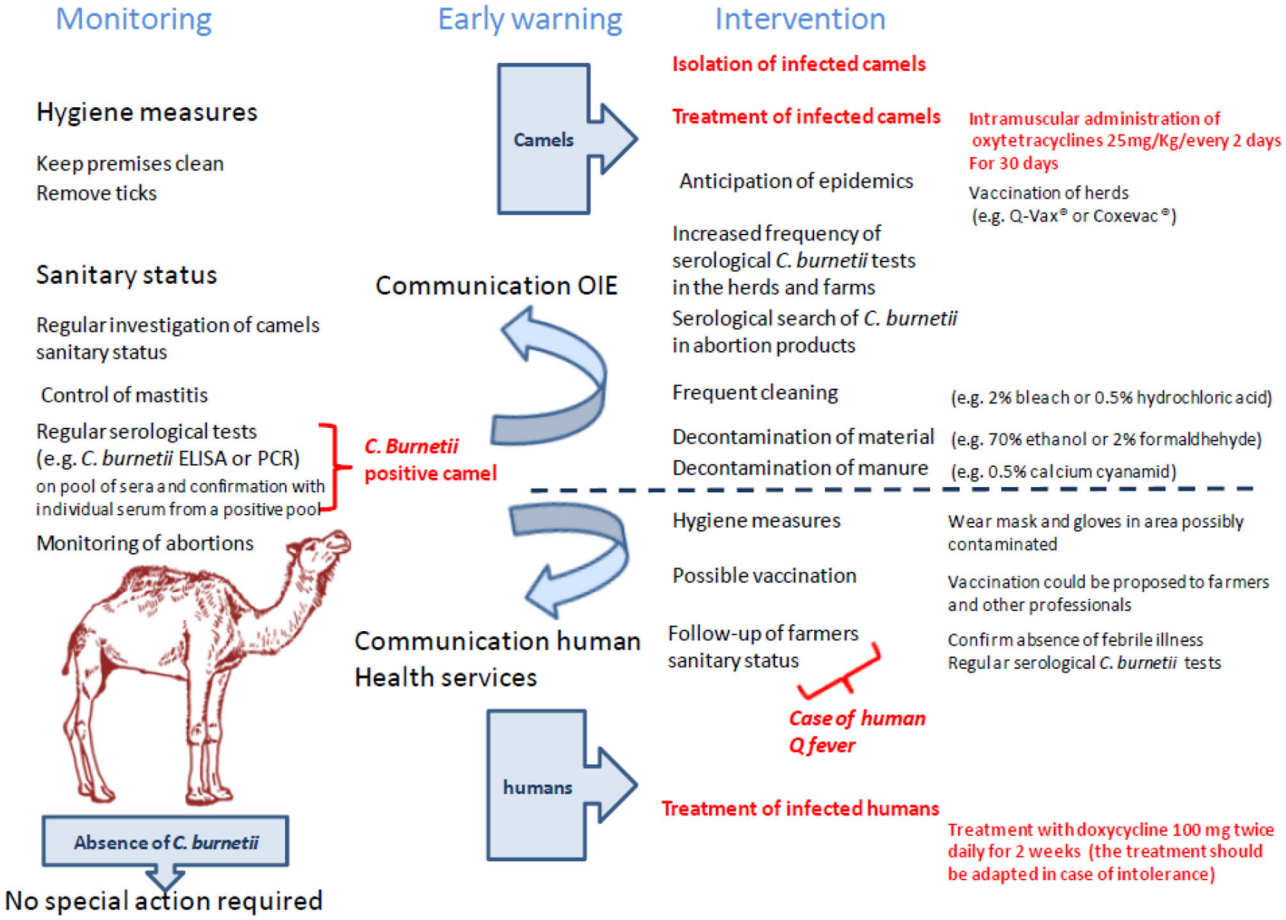
Suggested flowchart to monitor farms against the risk of *C. burnetii* infection. This logigram takes into account the surveillance, the alert, and the measures to be taken in case of presence of *C. burnetii*–infected animals in a herd. With regard to the specific actions to be implemented, they relate to both general safety measures and specific measures for sick camels or herds with sick animals and farmers.

The attitude to be adopted in case of contamination of a camel herd by *C. burnetii* did not drastically differ from those already applied to the treatment of other ruminants (Figure 7). In veterinary medicine, oxytetracyclines (OTC) were reported to be effective in decreasing the number of abortions in ruminants without preventing bacterial shedding and transmission (187, 493–495). Veterinarians recommended that cow with metritis or abortion be isolated and treated by parenteral antibiotic injections and intrauterine injections of OTC (8 mg OTC/kg/day for 30 days). The female must then be vaccinated and may later be inseminated (493). Yet, OTC treatments do not guarantee the elimination of bacteria from the milk of infected female (496). In France, when *C. burnetii* is detected in a herd of ruminants, sale of milk and transformation into cheese of milk from aborted female are strictly forbidden. Milk of the remainder of the flock can be sold after pasteurization (72°C during 15 min) (497). A similar preventive strategy is used in small ruminants (498). Because OTC at a dose of 25 mg/kg administered intramuscularly every 2 days for 30 days was found effective in eliminating bacterial shedding in camels infected by *Brucella melitensis* (322), it suggests that a similar treatment could be used for the treatment of *C. burnetii* infection, although such therapy has not yet been evaluated in camels infected with *C. burnetii*.

To design an effective strategy of control and prevention, it is necessary to remember that *C. burnetii* is able to survive in the external environment for a long period under a pseudosporulated form. Survival was estimated 1 h at 60°C in milk; 5 months in the soil, 6 months in the dried blood, 24 months in tick waste, and 1 month in the dried milk and meat (494, 499–501), suggesting that the bacteria can be transmitted within camel herds, as well as to other livestock and humans. In farms, the prevention of transmission is not an easy challenge because *C. burnetii* is also resistant to conventional disinfectants such as 0.5% formalin, 1% phenol, and 0.5% hypochlorite (495); *C. burnetii* is nevertheless destroyed by 0.5% hydrochloric acid, chlorinated lime at 2%, 1% formalin, 5% hydrogen peroxide, and 2% bleach (502, 503). In the Netherlands, spread of manure (feces) from infected herds is forbidden for at least 3 months after suspicion of infection (52). Decontamination of feces from infected animals is possible by adding 0.5% of calcium cyanamide to contaminated dung (504). Decontamination of surface and materials can be performed using 2% formaldehyde, 5% hydrogen peroxide, 70% ethanol, or 5% chloroform (439).

## Discussion

The Mediterranean population has experienced a growth rate of 20% between 1970 and 2019. In 2016, the number of inhabitants living in the 22 riparian countries of the Mediterranean basin was estimated 502 million people (7% of the world population). According to World Bank, the population of this region will reach 524 million inhabitants by 2025. North Africa, with a population of almost 200 million people (about 36 million inhabitants in Morocco, 43 million in Algeria, 12 million in Tunisia, 6.5 million in Libya, 97.5 million in Egypt) in 2019, represents about 40% of the population of all the Mediterranean basin, and this is where the population growth is fastest. During the period 1965–2000, the urban population growth rate increased by 3.76, 2.96, 2.82, and 2.26 per year in Libya, Algeria, Morocco, and Tunisia, respectively. Improvement of standards of living combined with a high population growth rate and rapid urbanization has caused a massive increase in demand of livestock products that native breeds could not satisfy. This situation resulted in massive food importation and intensification of livestock production systems. So far, the food of the inhabitants of North Africa was based on farms quite similar to those found in the Northern regions of the Mediterranean basin, especially dairy cattle and poultry. Until recently, little was invested in dromedary camel production development. Nowadays, dromedary camel breeding becomes an issue to face the growing food demand of North Africa.

*C. burnetii* was first reported in Africa in the late 1940s (157, 505). Until recently, *C. burnetii* serosurvey focused mainly on goats, sheep, and cattle, probably because in Australia, America, and Europe, where *C. burnetii* outbreaks in herds have produced significant economic losses and epidemics in humans, there are hardly any camels. Yet, in the North Africa and Arabic peninsula, dromedary camels could become the most important reservoir of *C. burnetii* in ruminants. This hypothesis is supported by the fact that in the past decade the camel seroprevalence was always the highest among all ruminants. In this area of the world, close contacts with dromedary camels were associated with increased *C. burnetii* seropositivity in humans (95). For the moment, dromedary camels represent <3% of livestock in countries where camels live. However, if governments decide a sharp rise in dromedary camel production, it will become urgent to better control the sanitary status of these animals and to implement breeding methods to protect herds from epizooties and humans from *C. burnetii* zoonoses.

As shown in Table 4, when dromedary camel seroprevalences for anti–*C. burnetii* Ig are compared over time, it appears that there are many more seropositive animals today than in the past. This could indicate either a strong progression in dromedary camel seroprevalences to *C. burnetii* in recent decades or that a recent increase in the number of serological test performed or the improvement of the diagnosis methods has artificially suggested a progression while the seroprevalence in camels has always been very high. Even if a change in the frequency of serosurvey or in diagnosis methods has allowed a better evaluation of *C. burnetii* occurrence in herds, it cannot introduce an experimental bias accounting for the highest seroprevalence for *C. burnetii* found in camels among ruminants. An important information that is generally absent from the publications on the subject concerns the period of collection of samples for which it is not known whether they have been taken during an outbreak or not. Likewise, it remains difficult to estimate the accuracy of the serological tests in camels, and it should be borne in mind that antigenic cross-reactions between *C. burnetii* and other bacteria are possible and have been reported for *Legionella pneumophila* (506). This could introduce a bias into these studies without, however, influencing the relative percentages of seroprevalence within the different species studied in the same series. The serosurveys by Schelling and colleagues in Chad, Hussein and colleagues in Sudan, and Klemmer and colleagues in Egypt are representative of this disturbing finding. In Chad, the anti–*C. burnetii* Ig seroprevalence was 80% in camels, 33% in sheep, 23% goats, and 4% in cattle; in Sudan, the seroprevalence was 64.3% in camels and 29.9% in cattle; in Egypt, the seroprevalence was 40.7% in camels, 19.3% in cattle, 8.9% in sheep, and 6.8% in goat. This intriguing high rate of *C. burnetii* infection in camel herds compared to other ruminants could be attributed to a number of factors, yet none was demonstrated so far to account for the high seroprevalence in camels. Because camels are known to show a high rate of ticks infestation (>99%) (507), and because ticks were found to carry *C. burnetii* and to transmit the pathogen (401, 402), it has been hypothesized that ticks widespread in the Sahara and North Africa could be vectors of *C. burnetii* among camels (378). It could fit with the observation that the highest rate of *C. burnetii* infection in camel herds was in old animals that have had more chance to be bitten by ticks in their life. However, this does not easily agree with the observation that female had higher seroprevalence than male (378). It is also difficult to determine whether these high seroprevalences are related to spreads of the pathogen in local herds or to increased transmission through contacts with animals imported for commercial purposes. Whatever the reasons, the dromedary camel could be the first source of *C. burnetii* among ruminants in countries of the Southern coast of the Mediterranean basin.

The incidence of abortion due to *C. burnetii* in animal herds in African ruminants remains to be better documented. In Morocco, *Chlamydia abortus* and *C. burnetii* were found associated with abortions of small ruminants, causing a significant economic loss (508, 509). A survey in Rabat reported that sheep with history of abortions were more likely to be seropositive for *C. burnetii* than those with normal births, 33% vs. 15%, respectively (510). More recently, a survey of abortion highlighted an average abortion of 12.1% in ewes, and the serological analysis of sheep and goat indicated that 57% were seropositive for *C. burnetii*, their seroprevalence being 91% for *Chlamydia*, 74% for *Toxoplasma*, 43% for *Brucella*, and 22% for *Leptospira*, respectively (510). To our knowledge, *C. burnetii*–induced abortion in dromedary camels was not evaluated so far.

The measures to be held for prevention, treatment, and control of zoonoses are usually available to humans and livestock in high-income countries but less present in low-income settings, which are the most vulnerable (511). Although dromedary camels can be suspected to play a major role in the spreading of *C. burnetii* to humans, their serosurvey remains rare. In addition, it is not known whether the camels found seropositive for *C. burnetii* still produce infectious bacteria or if the seroprevalence is simply the immunological signature of an old infection in animal that has gotten rid of the bacteria. Yet, the high prevalence of Q fever in camels, coupled with the widespread habit of consuming raw camel milk, underscores a possible role of camels in Q fever transmission to humans. If human Q fever cases have been documented in connection with consumption of raw milk from camels infected with *C. burnetii*, there are so far no reports of a human outbreak traced to camels. This does not mean that it does not exist especially in a context where it has not been systematically sought. Further research to evaluate the role of camels in *C. burnetii* transmission to humans should be assessed keeping in mind that infection with *C. burnetii* in humans is most often asymptomatic. Moreover, there is also a risk for people who come into close animal contact during mass gatherings like the annual Hajj pilgrimage to Saudi Arabia where more than 10 million Muslims from around 184 Islamic countries meet together. The overall correlation of camel numbers and human Q fever remains to be further explored. International cooperation and intersectorial governance are required for the control of *C. burnetii* zoonosis. Finally, these observations must raise physicians' awareness to the importance of notifying Q fever so that the real incidence may be found.

## Author Contributions

CD wrote the paper. All authors contributed to conceive the paper.

## Conflict of Interest

The authors declare that the research was conducted in the absence of any commercial or financial relationships that could be construed as a potential conflict of interest.
